# The CorC proteins MgpA (YoaE) and CorC protect from excess-magnesium stress and are required for egg white tolerance and virulence in *Salmonella*

**DOI:** 10.1128/mbio.02021-25

**Published:** 2025-08-18

**Authors:** Yumi Iwadate, James M. Slauch

**Affiliations:** 1 Department of Microbiology, University of Illinois at Urbana-Champaign, Urbana, Illinois, USA; University of Utah, Salt Lake City, Utah, USA

**Keywords:** magnesium, polyamines, *Salmonella*, cations, stationary phase

## Abstract

Cation homeostasis is a vital function. In *Salmonella*, growth in very low Mg^2+^ induces expression of high-affinity Mg²^+^ transporters and the synthesis of polyamines, organic cations that substitute for Mg²^+^. Once Mg²^+^ levels are re-established, the polyamines must be excreted by PaeA. Otherwise, cells lose viability due to a condition we term excess-cation stress. We sought additional tolerance mechanisms for this stress. We show that CorC and MgpA (YoaE) are essential for survival in stationary phase after Mg^2+^ starvation. Deletion of *corC* causes a loss of viability that is additive with the *paeA* phenotype. Deletion of *mgpA* causes a synergistic defect in the *corC* background. This lethality is suppressed by the loss of the inducible Mg^2+^ transporters, showing that the *corC mgpA* mutant is sensitive to changes in intracellular Mg^2+^. CorC and MgpA function independently of PaeA. A *paeA* mutant is sensitive to externally added polyamine in stationary phase, while the loss of CorC and MgpA suppressed this sensitivity. Conversely, the *corC mgpA* mutant, but not the *paeA* mutant, exhibited sensitivity to high Mg^2+^ and egg white. The *corC mgpA* mutant is also attenuated in a mouse model. The *corC* and *mgpA* genes are induced in response to increased Mg^2+^ concentrations. Thus, CorC and MgpA have overlapping roles in cation homeostasis, most probably acting to efflux excess Mg^2+^. Our data suggest that cells maintain relative concentrations of various cations that likely compete for binding to anionic components. The imbalance of these cations affects some essential function(s), leading to a loss of viability.

**IMPORTANCE:**

Mg²^+^ and other cations are critical for counteracting anionic compounds in the cell including RNA, DNA, and nucleotides. Both excessively low and excessively high cation levels are toxic. To maintain proper intracellular concentrations, cells must regulate Mg²^+^ importers and exporters or modulate the levels of other cations or anions that affect free Mg²^+^ levels. In *Salmonella*, no mutants sensitive to high Mg²^+^ levels have been identified. Here, we demonstrate that the largely uncharacterized proteins MgpA and CorC are induced under high Mg²^+^ conditions and are essential for tolerance to high Mg²^+^ levels. These genes are also essential for survival during endogenous excess-cation stress triggered by the transition to stationary phase after Mg²^+^ starvation, as well as for virulence, highlighting the broader role of cation homeostasis.

## INTRODUCTION

Cations, particularly magnesium (Mg^2+^), are required to stabilize RNA and DNA (1–3), counteract the negative charges on ATP (4), and neutralize the negative charges in outer membrane lipopolysaccharide (LPS) (5). Mg^2+^ is also critical for ribosome assembly, function, and stability (4, 6). Polyamines are organic cations important in all domains of life, but their physiological role is not well understood. Our recent data support a new paradigm for the overall role of polyamines in cell physiology. Our primary hypothesis is that polyamine and Mg^2+^ concentrations are coordinately controlled in the cell and that polyamines act as simple cations that can substitute for Mg^2+^ in critical systems under low-Mg^2+^ stress (7, 8).

**Fig 1:**
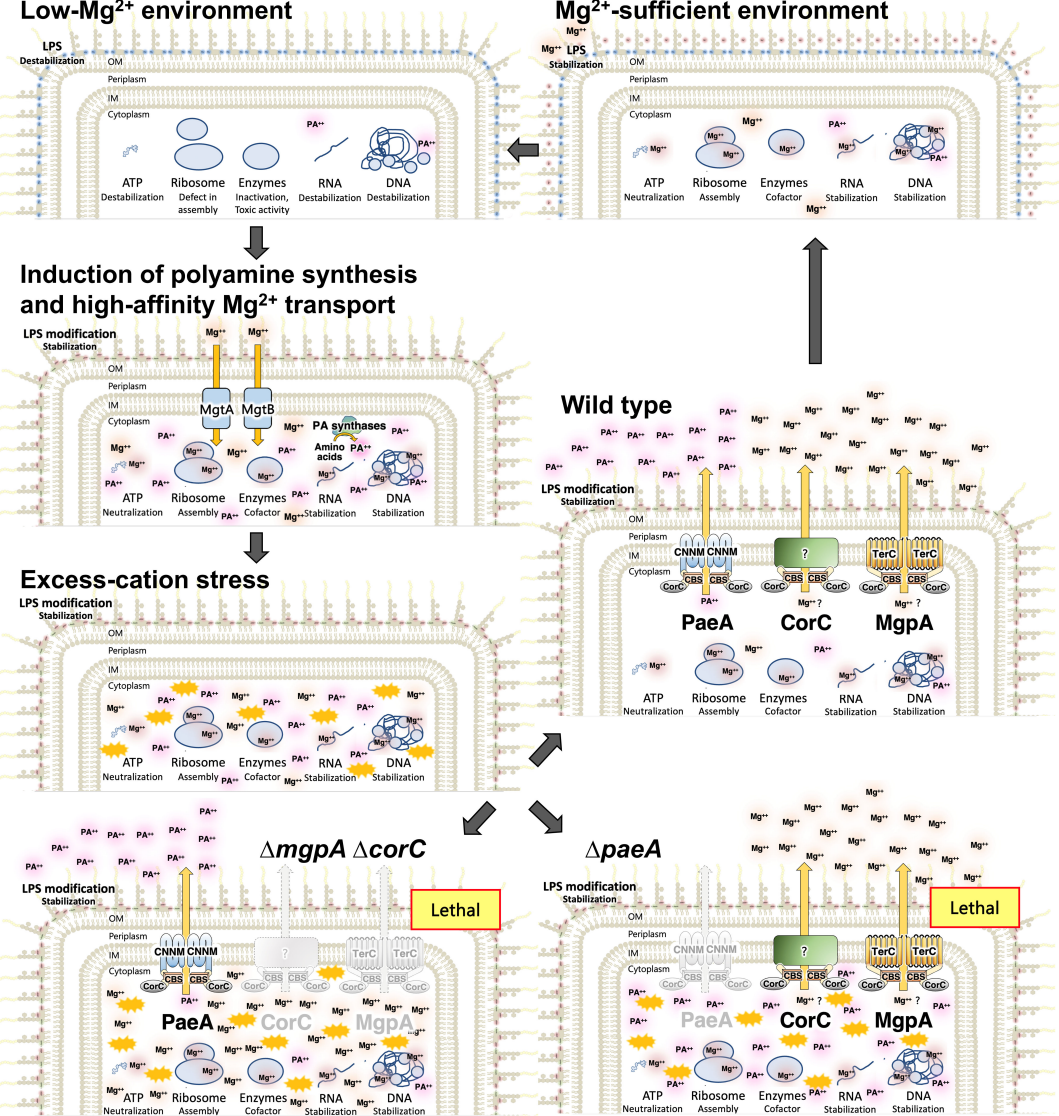
Model for excess-cation stress in *Salmonella*. Under Mg²^+^ starvation, *Salmonella* initially experiences low-cation stress. The cells induce polyamine synthesis to compensate for the Mg²^+^ deficiency and high-affinity Mg²^+^ transporters MgtA and MgtB to scavenge trace Mg²^+^ from the environment. Once intracellular Mg²^+^ levels are re-established, endogenous excess-cation stress arises. The CorC-domain-containing proteins PaeA and CorC/MgpA alleviate this stress by reducing intracellular polyamine and Mg²^+^ levels, respectively.

*Salmonella* Typhimurium is a major food-borne pathogen that replicates within macrophages, and adaptation to the low Mg^2+^ environment of the macrophage phagosome is key to *Salmonella* virulence. Counterintuitively, shifting *Salmonella* cells to Mg^2+^-depleted conditions induces an ultimate overload of cations that can be lethal upon reaching stationary phase. Upon Mg^2+^ starvation, polyamine synthesis is induced (7, 8), as is production of high-affinity Mg^2+^ transporters MgtA and MgtB (9–13) (Fig. 1). Either polyamine synthesis or Mg^2+^ transport is required to maintain viability under these low Mg^2+^ conditions. Once Mg^2+^ levels are re-established, the excess polyamines must be excreted by the inner membrane polyamine exporter PaeA (7, 14). Mutants lacking PaeA lose viability when cells reach stationary phase. The lethality of the *paeA* mutant in stationary phase is suppressed by blocking Mg^2+^ transport, indicating that it is the total concentration of Mg^2+^ and polyamines that is detrimental, a phenomenon that we term “excess-cation stress.” Importantly, these results are recapitulated during infection. Polyamine synthesis mutants are attenuated in a mouse model of systemic infection, as are strains lacking the MgtB Mg^2+^ transporter. Combined, these mutations confer a synergistic phenotype in animals, confirming that Mg^2+^ and polyamines are required for the same overall processes. These data support our hypothesis that the cell coordinately controls polyamine and Mg^2+^ concentrations to maintain overall cation homeostasis.

**Fig 2:**
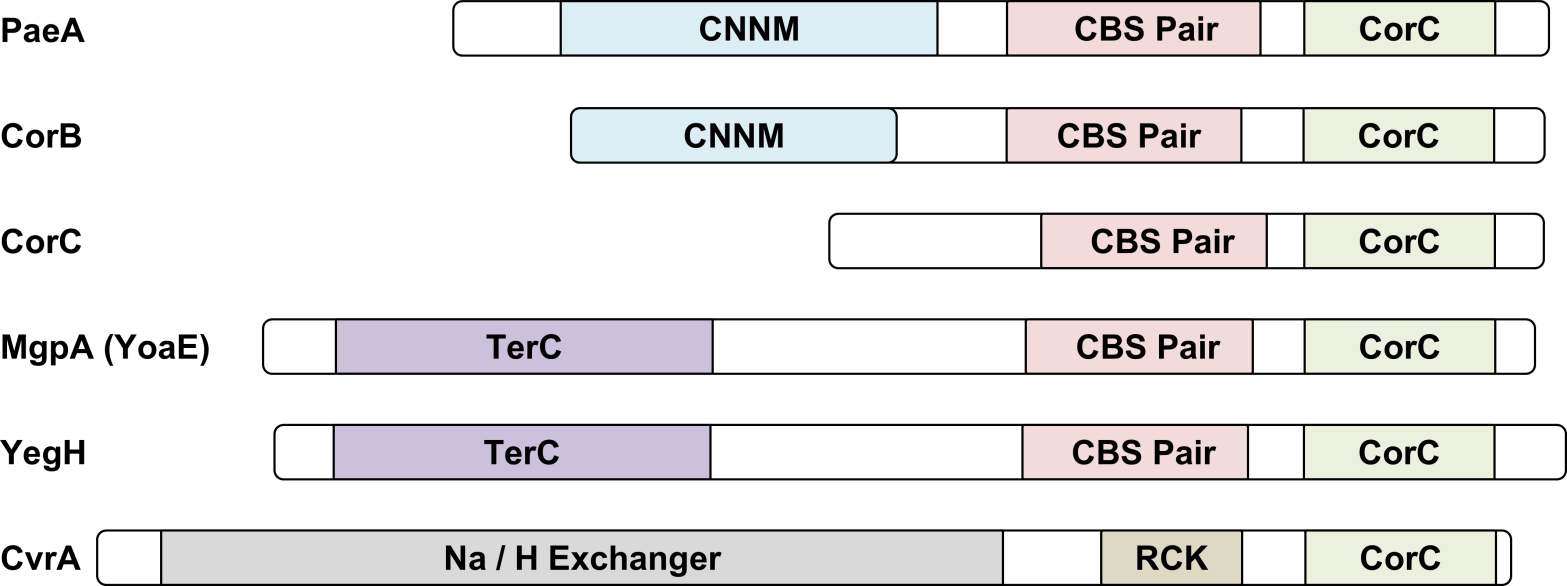
Domain architecture of CorC domain-containing proteins in *Salmonella.* Conserved domains were predicted using InterPro (https://www.ebi.ac.uk/interpro/). Each domain is represented by a different color, while regions with no domain assignment are shown in white. Key domains include cytoplasmic CorC_HlyC domain (pfam03471; function unknown; associated with ion transporters), transmembrane CNNM domain (pfam01595; found in cation transporters), cytoplasmic CBS-pair domain (pfam00571; shown to dimerize with Mg^2+^-ATP at the interface), transmembrane TerC domain (pfam03741; discovered in tellurite resistance proteins), transmembrane Na^+^/H^+^ exchanger domain (pfam00999), and cytoplasmic RCK domain (pfam02080; also associated with Na/H antiporters).

We sought additional proteins involved in mitigating excess-cation stress in *Salmonella*. We focused on the domain structure of the PaeA protein, particularly its C-terminal CorC domain. The PaeA protein contains an N-terminal transmembrane CNNM domain (pfam01595), found in cation transporters (15); a cytoplasmic portion containing a CBS-pair domain (pfam00571), shown to dimerize with Mg^2+^-ATP at the interface (16); and a C-terminal CorC_HlyC domain (pfam03471) of unknown function. The *Salmonella* genome encodes five additional proteins with CorC domains: CorB, CorC, MgpA (YoaE), YegH, and CvrA (Fig. 2). CorB resembles PaeA with a CNNM transmembrane domain, CBS-pair domain, and C-terminal CorC domain. The eponymous CorC lacks a transmembrane domain and has only a CBS-pair domain and a C-terminal CorC domain. MgpA and YegH each possess an N-terminal transmembrane TerC domain (pfam03741), discovered in tellurite-resistance proteins, followed by a CBS-pair domain and a C-terminal CorC domain. The CvrA protein contains an N-terminal transmembrane Na/H^+^ exchanger domain (pfam00999) and a cytoplasmic RCK domain (pfam02080), also associated with Na/H antiporters, followed by a CorC domain.

Although widespread, the biological and biochemical functions of the CorC domain remain unknown. We postulate that the CorC domain or the CBS-pair/CorC domains function as a sensor of cytoplasmic cations, such as Mg^2+^. For example, our model suggests that PaeA exports polyamines in response to increasing Mg^2+^ concentration (7). We hypothesize that Mg^2+^or Mg^2+^-ATP (16) bound to the CBS-pair/CorC domains activates polyamine efflux. Moreover, all characterized CorC domain-containing proteins appear to be involved in cation transport. PaeA in *Salmonella* and *E. coli* is responsible for the efflux of cadaverine and putrescine (14). CorC and CorB were originally identified as conferring resistance to cobalt in the medium and were further implicated in Mg^2+^ efflux (17). CorB homologs in the thermophilic archaeon *Methanoculleus thermophilus* and the thermophilic Gram-negative bacterium *Tepidiphilus thermophilus* are well-characterized Mg^2+^ efflux transporters (16, 18). In *Staphylococcus aureus*, the CorB homolog, MpfA, is essential for tolerance to high Mg^2+^ levels (19). The CvrA protein in *Vibrio cholerae* facilitates K^+^/H^+^ exchange (20). Collectively, these reports suggest that CorC domain-containing proteins are crucial for maintaining proper cation levels and relative composition.

Given these findings, we examined whether any of the CorC domain-containing proteins in *Salmonella* play a critical role in reducing excess-cation stress. Our results show that two of these proteins, CorC and MgpA (YoaE), are required for full survival during stationary phase after Mg^2+^ starvation, for full virulence in a mouse model, and for tolerance to high Mg^2+^ stress. Expression of both CorC and MgpA is also induced in response to increased Mg^2+^ in the medium. We have renamed *yoaE* to *mgpA*, for Mg^2+^ protection protein A. Thus, both CorC and MgpA play roles in mitigating excess-cation stress, whether it arises endogenously or exogenously, most likely by controlling excess Mg^2+^ efflux. Moreover, CorC and MgpA are required for egg white tolerance, suggesting that exposure to egg white imposes cation imbalance in *Salmonella*. Our findings reveal that multilayered systems involving CorC domain-containing proteins maintain proper cation levels in response to environmental changes, including during host infection.

## RESULTS

### CorC is required for full survival under excess-cation stress in stationary phase

**Fig 3:**
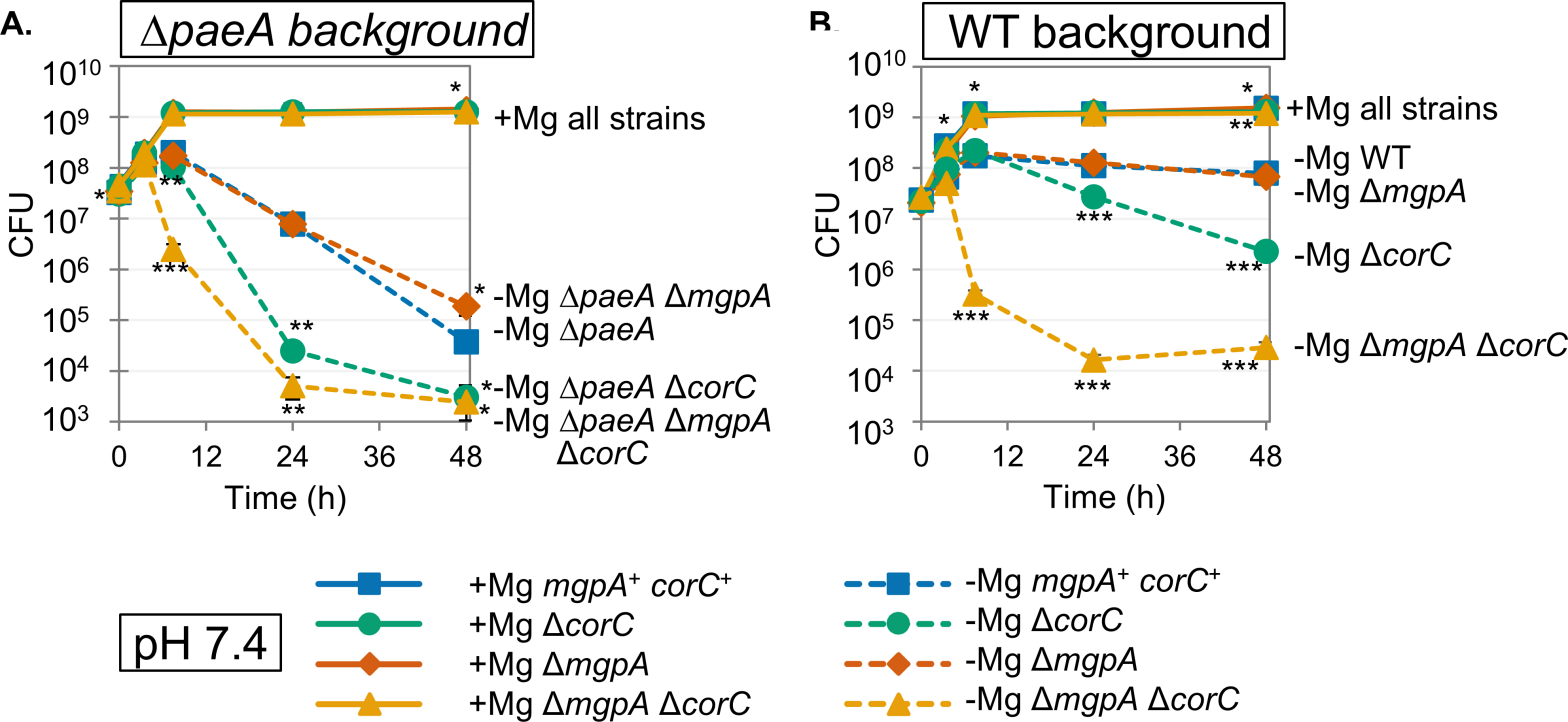
The ∆*corC* strain loses viability in stationary phase after Mg^2+^ starvation, and the deletion of *mgpA* exacerbates the effect. The indicated strains were grown overnight in N-minimal medium pH 7.4 with 10 mM MgCl_2_, washed, diluted into N-minimal medium pH 7.4 with or without 10 mM MgCl_2_ (t = 0 h), and incubated at 37℃. CFUs were determined at the indicated time points in ∆*paeA* (**A**) and wild-type (**B**) background. Values are mean ± SD, *n* = 6. Unpaired *t-*test (*P* < 0.05*, 0.005**, 0.0005***) vs. *mgpA*^+^ *corC*^+^ parent strain at the same time point and at the same Mg²^+^ concentration. Strains used: 14028, JS2692, JS2693, JS2694, JS2695, JS2696, JS2697, and JS2698.

Our previous studies revealed that, upon Mg^2+^ starvation, *Salmonella* induces both polyamine synthesis and high-affinity Mg^2+^ transport. Once Mg^2+^ levels are restored, PaeA is responsible for the efflux of the polyamines cadaverine and putrescine. In the absence of PaeA, cells start to die upon entry into stationary phase (7, 14) (Fig. 3A). We seek to further understand this excess-cation stress in stationary phase. The C-terminus of PaeA contains a CorC domain. Although the function of the CorC domain remains elusive, it likely functions as a sensor for intracellular cations. We, therefore, hypothesized that other CorC domain-containing proteins could also contribute to alleviating excess-cation stress in stationary phase. We initially focused on the eponymous CorC protein.

The wild-type and ∆*corC* strains were grown in N-minimal medium (pH 7.4) with or without added Mg^2+^ at 37℃, and viability and OD_600_ were monitored for 48 hours. The ∆*corC* strain displayed a decline in viability upon entry into stationary phase after Mg^2+^ starvation (Fig. 3B). The OD_600_ was unaffected in the ∆*corC* strain compared to the wild type grown in no Mg^2+^, indicating that the cells were not lysing (Fig. S1A). The *corC* gene is transcribed in an operon with upstream genes *ybeZ* and *ybeY* and downstream gene *lnt* (21–23). To confirm that the phenotype results from loss of CorC, we introduced low-copy number plasmids pWKS30 (vector) or pWKS30-*corC* into the ∆*corC* strain and examined viability after Mg^2+^ starvation. As shown in Fig. S2A, the phenotype of the ∆*corC* strain was fully complemented by pWKS30-*corC*. These results suggest that CorC has an important role in maintaining viability in stationary phase after Mg^2+^ starvation, similar to PaeA.

### Loss of MgpA confers synergistic phenotypes in the ∆*corC* background

Although the biochemical function of CorC is unknown, phenotypes conferred by loss of CorC have been characterized (17, 23, 24). For example, a ∆*corC* strain exhibits reduced Mg²^+^-efflux activity compared to the wild type (17). However, CorC is predicted to be a cytoplasmic protein with no transmembrane domain, suggesting that CorC is not a transporter *per se*. Rather, CorC might regulate the activity of associated transporters. To investigate the mechanisms underlying the role of CorC in maintaining survival in stationary phase after Mg^2+^ starvation, we asked if there was any genetic interaction between CorC and other CorC domain-containing proteins.

The genes encoding the five other CorC domain-containing proteins were deleted in the wild-type and *corC* backgrounds. Survival of the mutants during stationary phase was examined in Mg²^+^-sufficient and Mg²^+^-deficient media. As we reported previously (7), a ∆*paeA* strain showed a loss of viability in stationary phase after Mg^2+^ starvation (Fig. 3A). The deletion of the *corC* gene in the ∆*paeA* background further decreased viability in stationary phase, with the results suggesting an apparent additive effect in the double mutant (compare Fig. 3A and B). This suggests that *paeA* and *corC* are independently involved in the same phenomenon, in a broad sense.

Deletion of *mgpA* had no apparent effect in stationary phase after Mg^2+^ starvation. However, deletion of *mgpA* in the *corC* background notably intensified the loss of viability upon transition to stationary phase after Mg^2+^ starvation (Fig. 3B). This suggests a synergistic relationship between the *mgpA* and *corC* genes, implying a partially redundant function for the two gene products. To confirm that the loss of viability in the stationary phase is indeed caused by the inactivation of both *corC* and *mgpA*, we performed a complementation test. Introducing the pWKS30-*corC* plasmid into the ∆*mgpA* ∆*corC* strain restored viability in the stationary phase, while a pWKS30-*mgpA* plasmid restored the phenotype to that of the ∆*corC* strain (Fig. S2B). StyR-291, a sRNA encoded downstream of *mgpA* and antisense to the 3’- terminus of the *pdeD* gene (25), does not affect the phenotype. A ∆*mgpA* ∆*corC* strain harboring the pWKS30-*mgpA-* StyR-291 plasmid showed the same level of suppression of the strain harboring pWKS30-*mgpA* (Fig. S2B). These results show that both CorC and MgpA are critical for survival in stationary phase after Mg^2+^ starvation.

To determine whether the exacerbating effect of *mgpA* deletion in the ∆*corC* strain is specific to *corC* or also occurs in the ∆*paeA* background, we examined the impact of *mgpA* deletion on the survival of the ∆*paeA* strain during stationary phase after Mg²^+^ starvation. Deleting *mgpA* had no phenotypic effect in the ∆*paeA* strain, showing that there is no genetic interaction between *mgpA* and *paeA* (Fig. 3A). The ∆*mgpA* mutation did exacerbate the phenotype seen in the ∆*corC* ∆*paeA* background, as expected (Fig. 3A). In contrast to *mgpA*, deletion of the remaining CorC domain-containing proteins, *corB*, *yegH*, *or cvrA*, conferred no apparent phenotype in either the wild-type or ∆*corC* backgrounds (Fig. S3), indicating that they are not involved in this CorC-related phenomenon under these conditions. Note that YegH has the same general domain structure as MgpA (Fig. 2). Together, these data show that CorC, MgpA, and PaeA all play a role in protecting against excess-cation stress in stationary phase. CorC and MgpA are synergistic, implying redundant functions, but both are additive with PaeA, implying that PaeA is mechanistically separate.

### Blocking Mg²^+^ import suppresses lethality in the ∆*mgpA* ∆*corC* background

Both high-affinity Mg²^+^ transport and polyamine synthesis are induced in response to low-Mg²^+^ stress (Fig. 1). Upon entry into the stationary phase, the polyamines must be exported; otherwise, cells lose viability. Therefore, the polyamine transporter PaeA is essential for survival in stationary phase following Mg²^+^ starvation. Lethality in the ∆*paeA* background can be suppressed by blocking polyamine synthesis (∆synth) or by blocking Mg²^+^ transport via deletion of *mgtA* and *mgtB*. Thus, it is the combined action of polyamines and Mg²^+^ that causes excess-cation stress, which endogenously arises in stationary phase after Mg²^+^ starvation (7).

**Fig 4:**
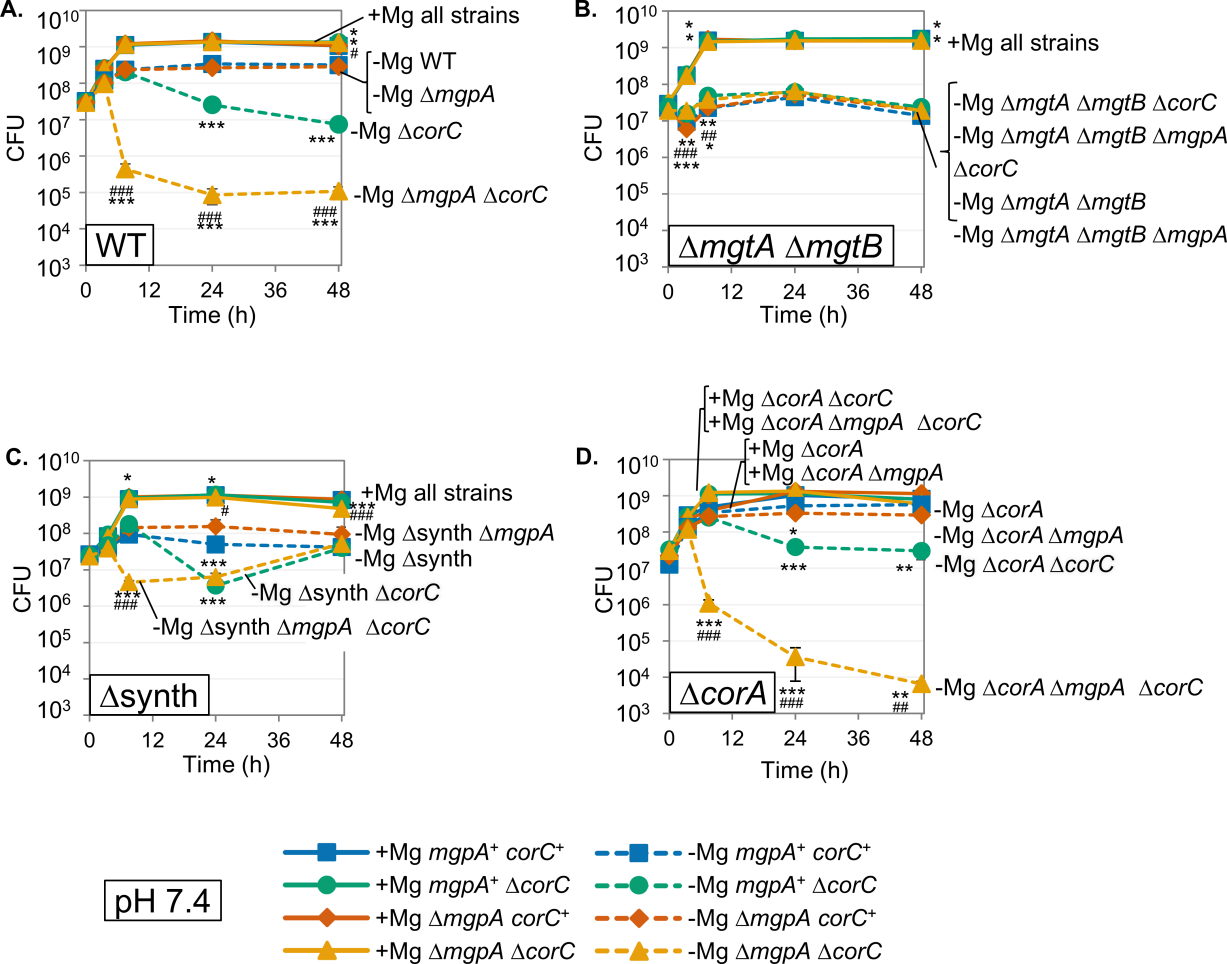
Loss of the high-affinity Mg²^+^ transporters MgtA and MgtB, but not CorA, suppresses the *corC* phenotype in stationary phase after Mg²^+^ starvation. The indicated strains were grown overnight in N-minimal medium pH 7.4 with 10 mM MgCl_2_, washed, diluted into N-minimal medium pH 7.4 with or without 10 mM MgCl_2_ (*t *= 0 h), and incubated at 37℃. CFUs were determined at the indicated time points in WT (**A**), ∆*mgtA* ∆*mgtB* (**B**), ∆synth (**C**), and ∆*corA* (**D**) background. Values are mean ± SD, *n* = 6. Unpaired *t-*test (*P* < 0.05*, 0.005**, 0.0005***) versus the *mgpA^+^ corC*^+^ at the same time point and at the same Mg²^+^ concentration, and (*P* < 0.05^#^, 0.005^##^, 0.0005^###^) ∆*mgpA corC^+^* versus ∆*mgpA* ∆*corC* at the same time point and at the same Mg²^+^ concentration. Strains used: 14028, JS2692, JS2693, JS2694, JS2713, JS2714, JS2715, JS2716, JS2560, JS2717, JS2718, JS2717, JS2720, JS2721, JS2722, and JS2723.

To further examine the roles of CorC and MgpA, we determined whether the deletion of *corC* and/or *mgpA* affected stationary-phase survival in genetic backgrounds suppressed for excess-cation stress, specifically in strains lacking the Mg²^+^ transporters MgtA and MgtB or lacking the ability to synthesize polyamines (∆synth). Loss of MgtA and MgtB completely suppressed the loss of viability conferred by the deletion of *corC* or of both *mgpA* and *corC* in stationary phase (Fig. 4A and B), mirroring the effect seen in the ∆*paeA* mutant (7). This similarity implies that the transport of Mg^2+^ via inducible Mg^2+^ transporters serves as a primary factor contributing to the loss of viability in these various mutants, consistent with the concept that Mg^2+^ starvation induces Mg^2+^ uptake, leading to excess-cation stress (Fig. 1). In contrast, the deletion of polyamine synthesis genes only partially suppressed the phenotypes in the ∆*corC* and the ∆*mgpA* ∆*corC* mutants (Fig. 4C), differing from the complete suppression observed in the ∆*paeA* strain (7). These data suggest that, while PaeA reduces excess-cation stress by reducing cellular polyamine levels, CorC and MgpA have a distinct role. The simplest model is that CorC and MgpA are involved in efflux of excess Mg^2+^ (Fig. 1).

### Phenotypes conferred by loss of MgpA and CorC are affected by pH

**Fig 5:**
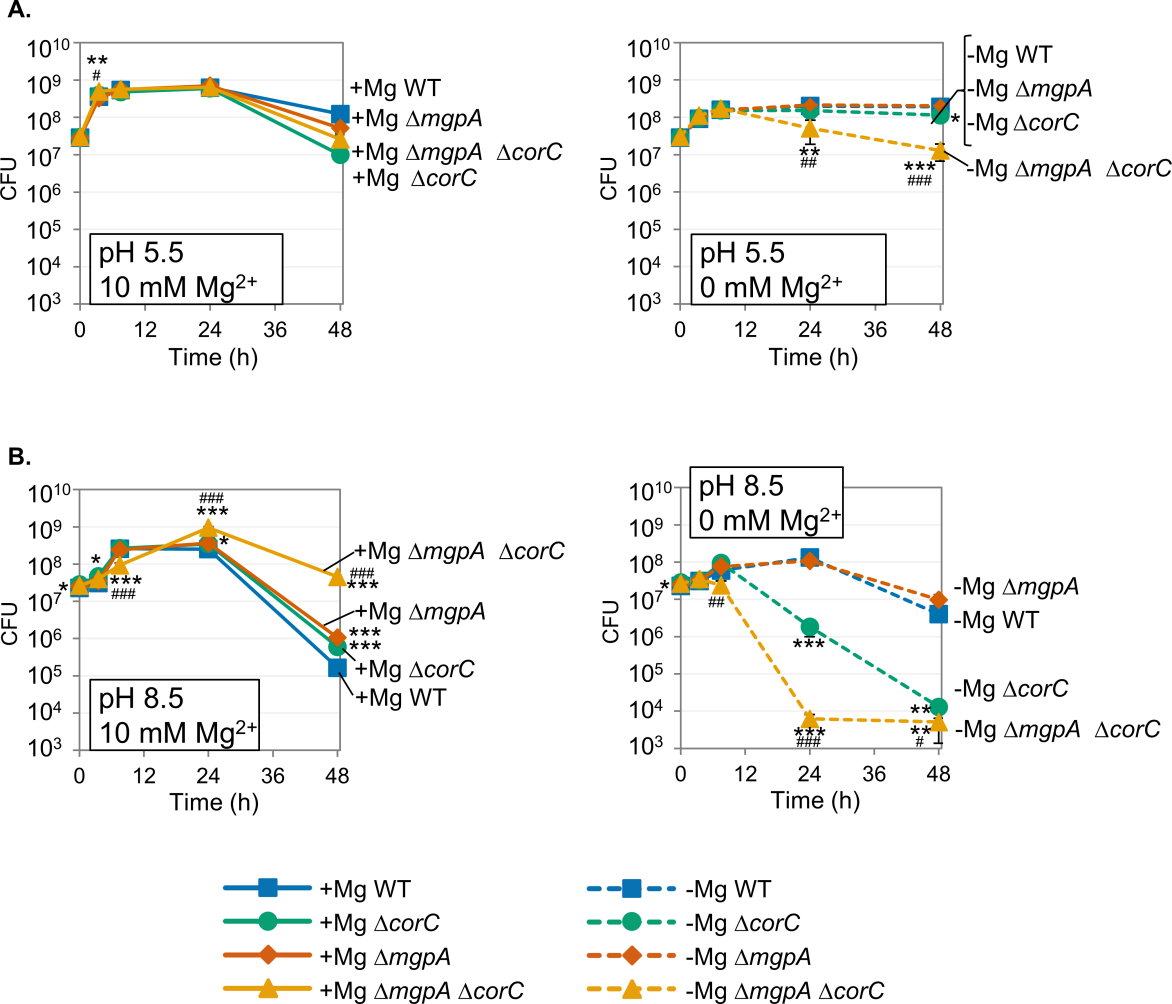
The loss of viability in ∆*corC* and ∆*mgpA* ∆*corC* strains during stationary phase after Mg²^+^ starvation is reduced in acidic medium and exacerbated in alkaline medium. The indicated strains were grown overnight in N-minimal medium pH 7.4 with 10 mM MgCl_2_, washed, diluted into N-minimal medium (**A**) pH 5.5 and (**B**) pH 8.5 with or without 10 mM MgCl_2_ (*t* = 0 h), and incubated at 37℃. CFUs were determined at the indicated time points. Values are mean ± SD, *n* = 6. Unpaired *t-*test (*P* < 0.05*, 0.005**, 0.0005***) versus corresponding WT and (*P* < 0.05^#^, 0.005^##^, 0.0005^###^) ∆*mgpA corC^+^* versus ∆*mgpA* ∆*corC* at the same time point and at the same Mg²^+^ concentration. Strains used: 14028, JS2692, JS2693, and JS2694.

Above, we tested the impact of deleting *mgpA* and/or *corC* on survival during stationary phase after Mg^2+^ starvation, specifically at pH 7.4. However, macrophages, for example, limit the growth of *Salmonella*, not only by restricting Mg^2+^ availability (26, 27), but also by acidifying the phagocytic vacuole (28, 29). To gain insight into the effect of pH on the phenotypes of the ∆*corC* and ∆*mgpA* ∆*corC* strains, we monitored the viability of wild-type, ∆*corC*, ∆*mgpA*, and ∆*mgpA* ∆*corC* strains after Mg^2+^ starvation when grown in acidic (pH 5.5) or alkaline (pH 8.5) N-medium. As shown in Fig. 5A, when grown in acidic medium, the ∆*corC* strain survived after Mg^2+^ starvation for 48 hours at the same level as the wild type. While the ∆*mgpA* ∆*corC* strain still exhibited a loss of viability in stationary phase when grown without Mg^2+^, the loss of viability was much reduced compared to that observed in neutral pH medium (Fig. 3B and 5A). In contrast, when grown in alkaline medium, both the ∆*corC* and ∆*mgpA* ∆*corC* strains exhibited a greater loss of viability in stationary phase after Mg^2+^ starvation compared to when grown in neutral pH medium (Fig. 3B and 5B). Altogether, the function of MgpA and CorC becomes more critical for survival in stationary phase after Mg^2+^ starvation as the pH rises. This could be an indirect effect in that the pH of the medium could affect transport of ions or induce additional homeostatic mechanisms.

### CorC functions independently of CorA

The *corC* gene was first identified through a screen for mutations conferring resistance to cobalt (17). In addition to *corC*, this screen identified *corB* and *corA*, the latter encoding the primary Mg^2+^ transporter in Mg-replete conditions, capable of both Mg^2+^ import and efflux (30). Gibson et al. (17) showed that a *corBCD* triple mutant, similar to the *corA* mutant, lost all apparent Mg^2+^ efflux activity. Therefore, even though *corC* and *corA* double mutants were never tested, it was suggested that *corC* and *corA* have a genetic interaction and that CorC requires CorA to function (17). To test if CorA is involved in the *corC* phenotype conferred in stationary phase after Mg^2+^ starvation, we examined the effect of deleting *corA* in the wild-type, ∆*corC*, ∆*mgpA*, and ∆*mgpA* ∆*corC* backgrounds. As shown in Fig. 4D, the loss of CorA had no significant effect on the ∆*corC* and ∆*mgpA* ∆*corC* phenotypes in stationary phase after Mg^2+^ starvation. Note that CorA does not function at low Mg^2+^ concentrations (31). Indeed, deletion of *corA*, *corB*, and *corD* had no effect on the *corC mgpA* phenotype (Fig. S4). These results show that CorC and MgpA function independently of CorA to alleviate endogenously induced excess-cation stress in stationary phase after Mg^2+^ starvation.

### Loss of CorC confers cobalt and manganese tolerance independent of MgpA

**Fig 6:**
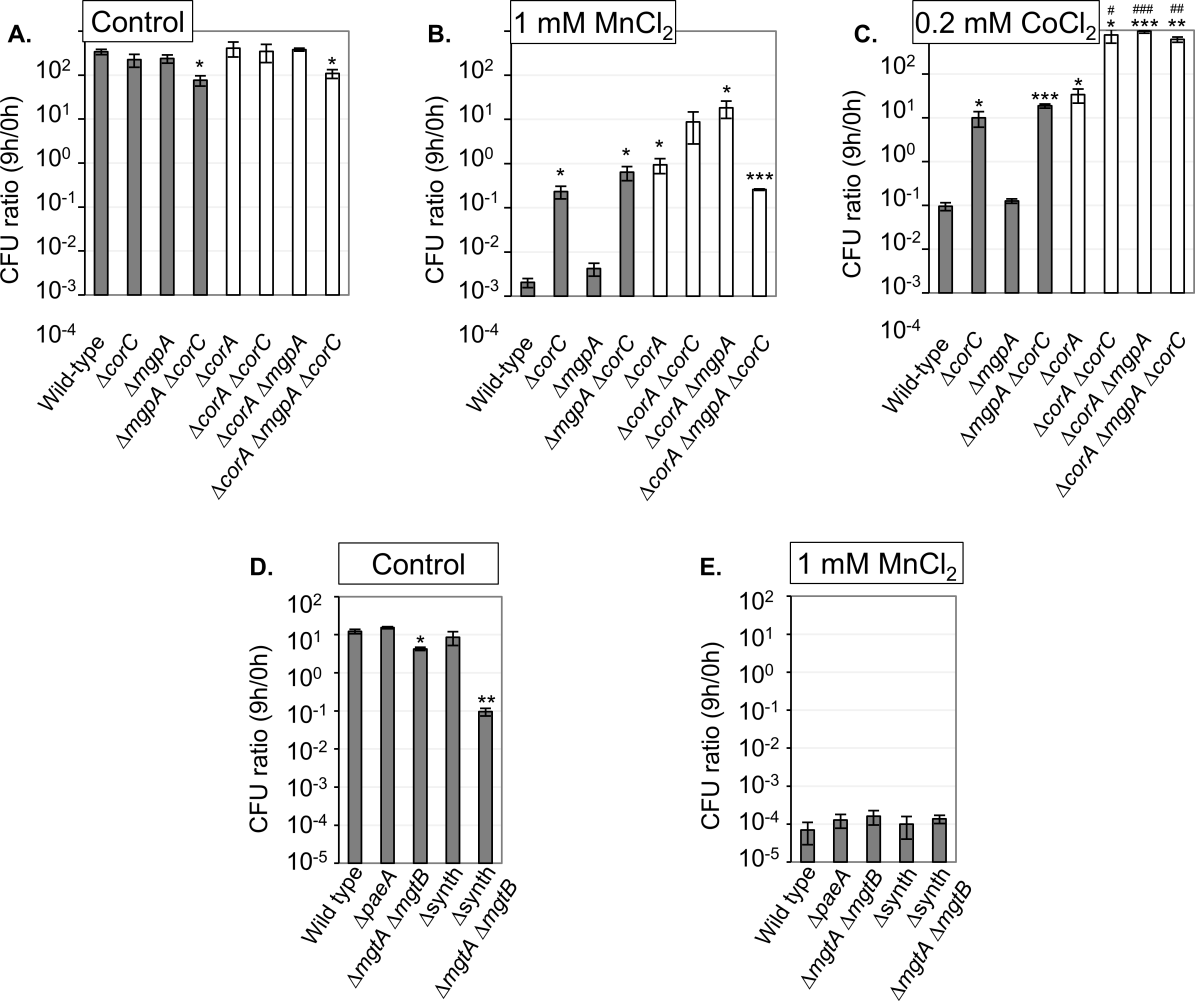
Loss of CorC, but not MgpA, increases tolerance to Co^2+^ and Mn^2+^ (A–E). The indicated strains were grown overnight in MOPS-minimal medium (pH 7.4) with 1.32 mM KH_2_PO_4_ and 10 mM MgCl_2_, washed, diluted into MOPS-minimal medium (pH 7.4) with or without the indicated amounts of MnCl_2_ or CoCl_2_ in the absence of KH_2_PO_4_ and MgCl_2_ (*t* = 0 h), and incubated at 37°C. CFUs were determined at 0 h and 9 h. CFU ratio (9 h/0 h) values are mean ± SD, *n* = 3. Unpaired *t-*test (*P* < 0.05*, 0.005**, 0.0005***) versus WT and (*P* < 0.05^#^, 0.005^##^, 0.0005^###^) versus ∆*corA*. Strains used: 14028, JS2692, JS2693, JS2694, JS2720, JS2721, JS2722, JS2723, JS2430, JS2562, JS2560, and JS2563.

As noted, *corC* mutants were originally selected as being resistant to cobalt (17). Resistance to Co^2+^ is correlated with resistance to Mn^2+^ in *Bacillus* (32). We examined the role of CorC and MgpA in Co^2+^ and Mn^2+^ tolerance. As shown in Fig. 6, deletion of *corC* increased tolerance to both Co^2+^ and Mn^2+^, similar to the phenotype conferred by deletion of *corA*, consistent with previous data (17). The double deletion of both *corC* and *corA*, which had not been examined in the previous study (17), further increased tolerance to Co^2+^ or Mn^2+^ compared to the single deletion mutants. However, the degree of increase suggests that CorC and CorA act additively and independently. These results further indicate that CorC does not require CorA to function. Interestingly, loss of MgpA had little effect on Co^2+^ or Mn^2+^ resistance in an otherwise wild-type or *corC* background, but it did enhance resistance in a *corA* background (Fig. 6A through C). Importantly, the ∆*paeA*, ∆*mgtA,* ∆*mgtB*, ∆synth, and ∆synth ∆*mgtA* ∆*mgtB* mutants showed similar tolerance to Mn^2+^ as the wild type (Fig. 6D and E), further showing that the role(s) of CorC and MgpA is distinct from that of PaeA. Altogether, these results confirm that loss of CorC somehow confers resistance to Co^2+^ or Mn^2+^. These results also show that CorC and MgpA act independently of each other with respect to this phenotype, and neither require CorA to function.

### The ∆*mgpA *and ∆*corC *mutants show higher tolerance to exogenous polyamines

The data above suggest that CorC and MgpA alleviate endogenously induced excess-cation stress after Mg²^+^ starvation, similar to the general role of PaeA, which functions specifically to efflux the divalent polyamines cadaverine and putrescine, whose synthesis is induced upon Mg²^+^ starvation (8, 14). Indeed, a ∆*paeA* mutant is sensitive to exogenous polyamines during stationary phase (14). CorC and MgpA appear to have functions distinct from that of PaeA, as blocking polyamine synthesis did not completely suppress the loss of viability in the ∆*corC* and ∆*mgpA* ∆*corC* strains—unlike in the ∆*paeA* strain (7)—after Mg²^+^ starvation (Fig. 4C). To further investigate the functional differences between CorC/MgpA and PaeA, we examined whether deletion of *corC* and/or *mgpA* conferred distinct phenotypes with respect to tolerance to exogenous excess-polyamine stress. We measured tolerance to exogenous polyamines after growth in medium with high or low Mg^2+^. We tested their phenotype in a ∆*paeA* background because this strain is sensitive to polyamines, and any additional effects should be easily detectable in this background. Strains were grown in medium with 50 µM MgCl_2_ (low Mg^2+^) or 10 mM MgCl_2_ (high Mg^2+^), washed, and incubated in a pH 8.5 buffer with the indicated polyamines. This level of low Mg^2+^ does not induce lethality in stationary phase. Note that high pH allows polyamines to be deprotonated and diffuse passively into the cell cytoplasm (14, 33).

**Fig 7:**
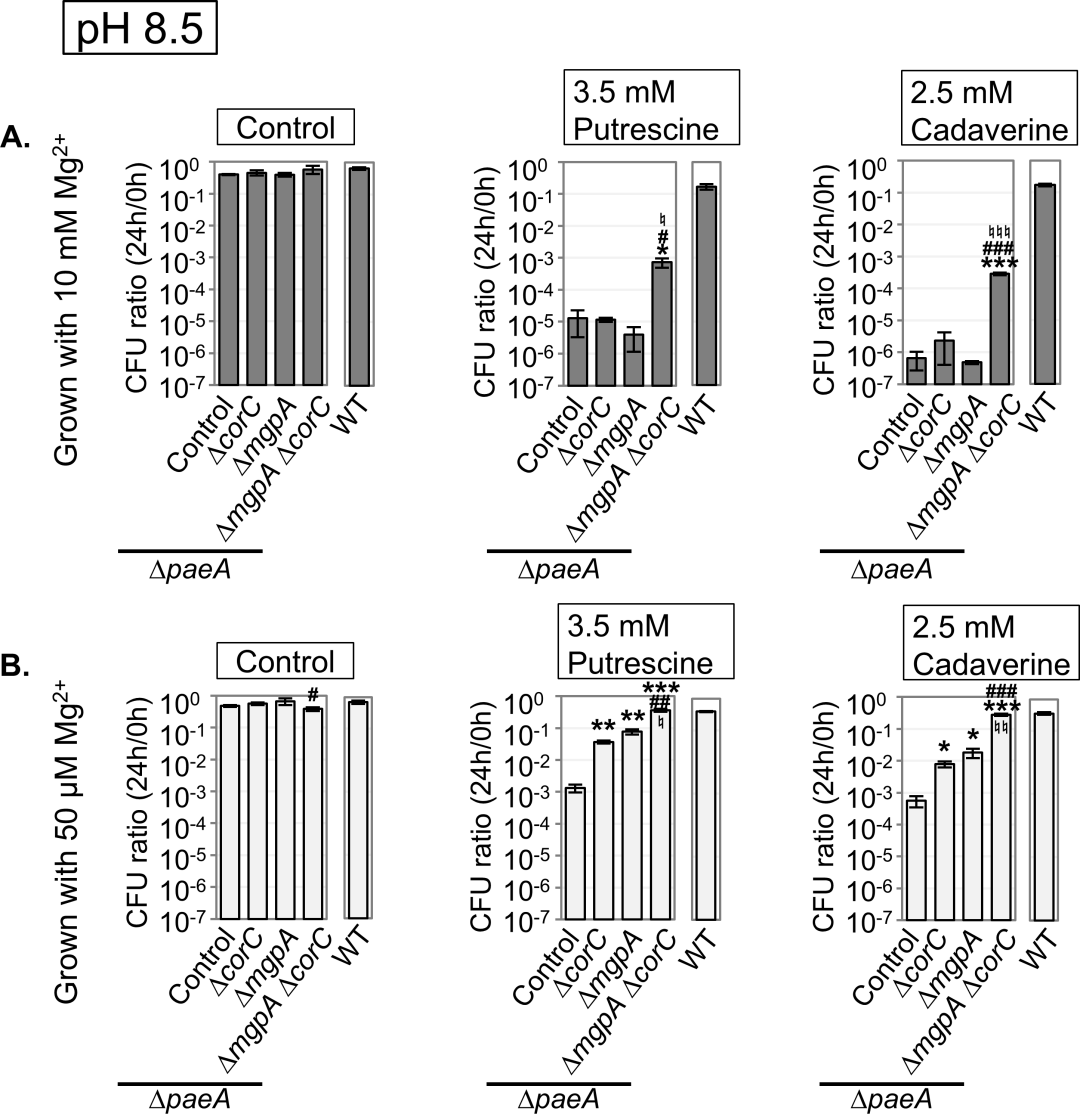
Loss of CorC and MgpA suppresses polyamine sensitivity in *paeA* background. The indicated strains were grown in N-minimal medium (pH 7.4) with (**A**) 10 mM or (**B**) 50 µM MgCl_2_ for 24 hours at 37°C, washed, diluted into pH 8.5-buffered saline containing indicated polyamines (*t *= 0 h), and incubated at 37°C. CFUs were determined at 0 hour and 24 hours. CFU ratio (24 h/0 h) values are mean ± SD, *n* = 3. Unpaired *t-*test (*P* < 0.05*, 0.005**, 0.0005***) versus WT, (*P* < 0.05^#^, 0.005^##^, 0.0005^###^) ∆*mgpA corC^+^* + ∆*mgpA* ∆*corC*, and (*P* < 0.05^♮^, 0.005 ^♮♮^, 0.0005 ^♮♮♮^) *mgpA^+^* ∆*corC* versus ∆*mgpA* ∆*corC*. Strains used: 14028, JS2695, JS2724, JS2725, and JS2726.

As expected, the *paeA* mutant, grown to stationary phase in either high or low Mg^2+^, was sensitive to either putrescine or cadaverine (Fig. 7). When cells were grown in low Mg^2+^, the sensitivity to polyamines was significantly reduced, as we have previously shown; it is the combined amount of Mg^2+^ and polyamines that is lethal (7). Deletion of both *corC* and *mgpA* did not further sensitize the *paeA* mutant to polyamines in both high or low Mg^2+^. Rather, sensitivity was suppressed. Deletion of either *corC* or *mgpA* individually in the *paeA* background had no effect when cells were grown in high Mg^2+^, but did suppress somewhat when cells were grown in low Mg^2+^ (Fig. 7). These results suggest that although CorC and MgpA alleviate endogenously induced excess-cation stress after Mg²^+^ starvation, similar to the general role of PaeA, they function through mechanisms distinct from PaeA, which directly reduces cytoplasmic polyamine levels. Indeed, we hypothesize that blocking Mg^2+^ efflux via deletion of *corC* and *mgpA* protects against high polyamine levels (see discussion).

### MgpA and CorC are required for tolerance to exogenous excess-Mg^2+^ stress

In our model (Fig. 1), endogenously induced excess-cation stress arises upon entry into stationary phase after Mg²^+^ starvation, due to the simultaneous activation of high-affinity Mg²^+^ transport and polyamine synthesis during growth phase. In the stationary phase, polyamine and Mg²^+^ levels exceed the cell’s requirements and cross some threshold level that is lethal for unknown reasons. Both PaeA and MgpA/CorC are required for survival under these conditions, yet they appear to have distinct functions, based on the above data. We hypothesized that MgpA and CorC are specifically required to maintain Mg²^+^ homeostasis during endogenously induced excess-cation stress.

**Fig 8:**
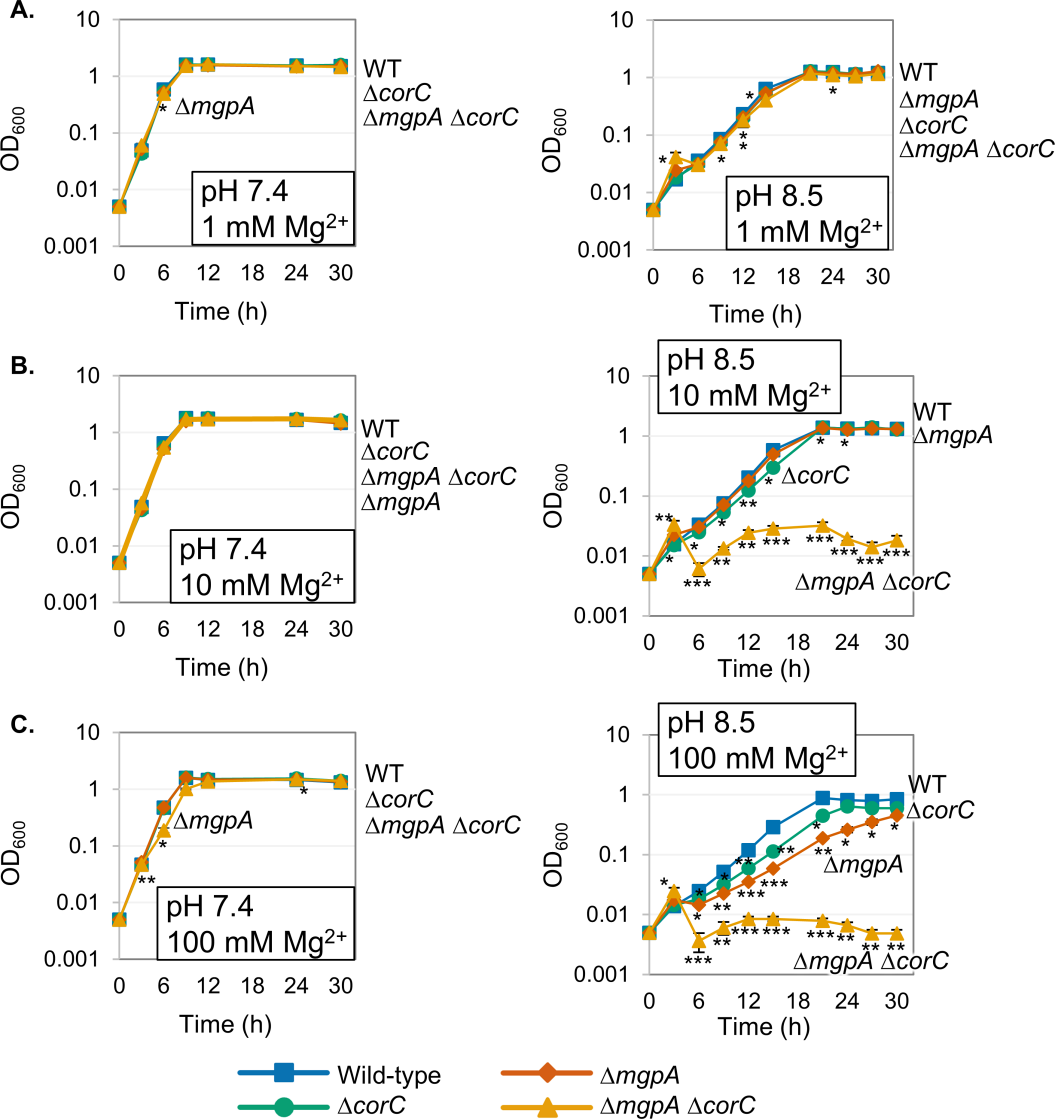
pH affects growth in high Mg^2+^ conditions. The indicated strains were pre-grown to mid-exponential phase in N-minimal medium pH 7.4 with 1 mM MgCl_2_, washed, and diluted into N-minimal medium pH 7.4 or pH 8.5 with (**A**) 1 mM, (**B**) 10 mM, and (**C**) 100 mM MgCl_2_ (*t* = 0 h), and incubated at 37°C. OD_600_ was determined at the indicated time points. Values are mean ± SD, *n* = 3. Unpaired *t-*test (*P* < 0.05*, 0.005**, 0.0005***) versus corresponding WT at the same timepoint. Strains used: 14028, JS2692, JS2693, and JS2694.

To test this hypothesis, the wild-type, ∆*corC*, ∆*mgpA*, and ∆*mgpA* ∆*corC* strains were pre-grown to the mid-exponential phase in pH 7.4 N-minimal medium with 1 mM MgCl_2_, then washed, diluted into pH 7.4 media with increasing amounts of MgCl_2_, and shaken vigorously at 37°C. OD_600_ and CFU were determined over 30 hours. When grown in pH 7.4 medium supplemented with 1, 10, or 100 mM MgCl_2_, all strains exhibited identical growth (Fig. 8A through C) and did not show any loss of viability (Fig. S5A through C). Since MgpA and CorA became more critical during endogenously induced excess-cation stress when grown in alkaline pH medium (Fig. 5B), cells pre-grown to the mid-exponential phase in pH 7.4 N-minimal medium with 1 mM MgCl_2_ were also diluted into pH 8.5 medium supplemented with 1, 10, or 100 mM MgCl_2_. When grown in pH 8.5 medium supplemented with 1 mM MgCl₂, all strains exhibited identical growth (Fig. 8A) and did not lose viability (Fig. S5A). When grown in alkaline medium supplemented with 10 mM or 100 mM MgCl₂, the ∆*mgpA* ∆*corC* strain ceased growth after 3 hours of incubation (Fig. 8B and C) and, at 100 mM Mg^2+^, showed a drastic loss of viability after 12 hours of incubation (Fig. S5C). This phenotype is synergistic; the single deletion mutants showed only minor phenotypes. The introduction of plasmid-borne *corC* or *yoaE* into the ∆*mgpA* ∆*corC* strain suppressed this high Mg sensitivity in the alkaline medium (Fig. S6), indicating that the inactivation of the *mgpA* and *corC* genes indeed is a cause of death by high Mg^2+^ in the alkaline medium. Collectively, these results show that the ∆*mgpA* ∆*corC* strain is sensitive to high Mg^2+^ in alkaline medium, consistent with our hypothesis that both MgpA and CorC are required to efflux excess Mg^2+^ to avoid excess-cation stress.

We also tested the roles of polyamines and PaeA under these conditions. Importantly, the ∆*paeA* strain did not exhibit any growth defects or loss of viability under high Mg^2+^ stress at pH 7.4 or 8.5 (Fig. S7 and 8), consistent with CorC and MgpA having functions distinct from that of PaeA. Interestingly, the strain incapable of synthesizing polyamines (∆synth) showed a severe growth defect at pH 8.5 compared to pH 7.4 (Fig. S7), but no loss of viability (Fig. S8). This defect was independent of Mg^2+^ concentration.

### The ∆*mgpA* ∆*corC* mutant accumulates Mg^2+^ in the cell

**Fig 9:**
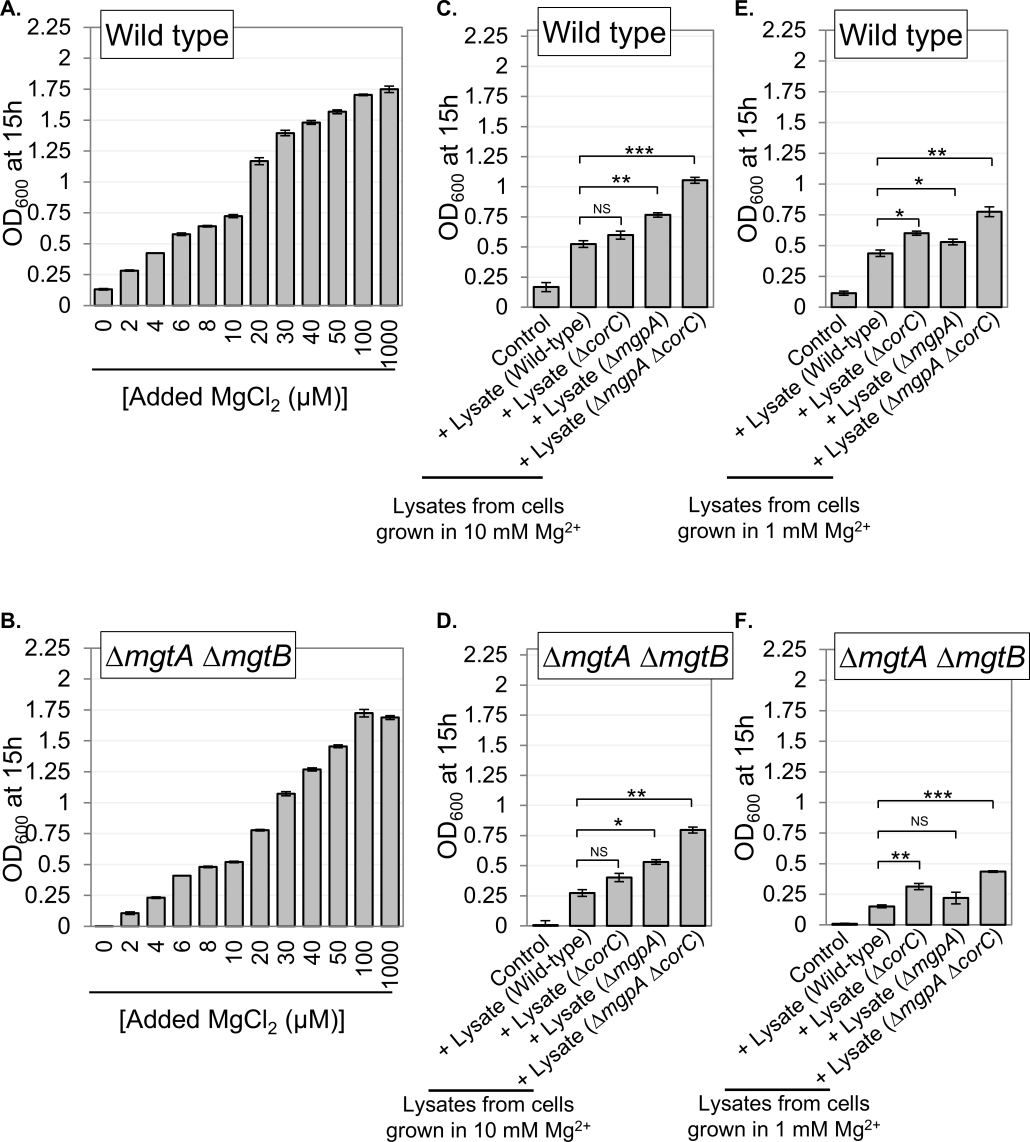
The lysate from the ∆*mgpA* ∆*corC* strain grown with Mg²^+^ stimulates the growth of both wild-type and ∆*mgtA* ∆*mgtB* strains in medium without added Mg²^+^ greater than the lysate from the wild type. The indicated strains were pre-grown to mid-exponential phase in N-minimal medium (pH 7.4) with 10 mM MgCl₂ at 37°C, washed, and then diluted into either (**A and B**) N-minimal medium (pH 7.4) with or without the indicated concentrations of MgCl₂, or (C–F) N-minimal medium (pH 7.4) with no added Mg²^+^, supplemented with 34  µg/mL of each lysate, followed by incubation at 37 ℃. Lysates were prepared from wild-type, ∆*corC*, ∆*mgpA*, and ∆*mgpA* ∆*corC* strains grown in the presence of (**C and D**) 10  mM or (**E and F**) 1  mM MgCl₂, as described in the Materials and Methods. Final OD₆₀₀ values were measured after 15 hours of incubation. Values are mean ± SD, *n* = 3. Unpaired *t*-test (*P* < 0.05*, 0.005**, 0.0005***) vs. *mgpA*^+^ *corC*^+^ parent at the medium. Strains used: 14028, JS2728, JS2729, JS2730, and JS2562.

The phenotypes conferred by loss of MgpA and CorC are clearly affected by Mg^2+^ levels in the medium. A simple model would suggest that MgpA and CorC either directly or indirectly efflux excess Mg^2+^. We indirectly quantified total intracellular Mg²^+^ levels in cells grown in high-Mg²^+^ medium using a bioassay, in which lysates prepared from wild-type, ∆*corC*, ∆*mgpA*, and ∆*mgpA* ∆*corC* strains were tested for their ability to stimulate the growth of wild-type and ∆*mgtA* ∆*mgtB* strains in medium lacking added Mg²^+^. Specifically, these strains were cultured in N-minimal medium (pH 7.4) supplemented with either 1 mM or 10 mM MgCl₂. None of the strains exhibited loss of viability at these Mg²^+^ concentrations (Fig. S9A and B). After 24 hours of incubation, cells were carefully harvested, washed, and lysates were prepared in Chelex-treated PBS. We then assessed the growth-stimulating activity of these lysates on wild-type or ∆*mgtA* ∆*mgtB* strains in N-minimal medium without added MgCl₂ by quantifying the final OD_600_ at 15 hours of incubation. In control experiments, added MgCl₂ increased the growth yield of both wild-type and ∆*mgtA* ∆*mgtB* strains in a concentration-dependent manner (Fig. 9A and B).

When lysates from wild-type cells grown in 10 mM MgCl₂ were added to the medium, the growth yield of both wild-type and ∆*mgtA* ∆*mgtB* strains increased by more than 2-fold (Fig. 9C and D). Lysates from the ∆*corC* strain stimulated growth to a similar extent as wild-type lysates. However, lysates from the ∆*mgpA* strain increased growth by approximately 1.5-fold more compared to the wild-type lysate, and lysates from the ∆*mgpA* ∆*corC* strain stimulated growth by more than 2-fold relative to the wild-type lysate. When lysates from cells grown with 1 mM MgCl₂ were used, wild-type lysates still stimulated growth yield in Mg²^+^-free medium, and ∆*mgpA* ∆*corC* lysates again stimulated greater growth yield than wild type, similar to the results with 10 mM-grown lysates (Fig. 9E and F). However, ∆*corC* lysates from cells grown with 1 mM MgCl₂ stimulated growth more than ∆*mgpA* lysates, which is the opposite of what was observed with lysates from cells grown in 10 mM MgCl₂. This suggests that *corC* activity may be more important when Mg²^+^ levels are moderate (e.g., 1 mM), whereas *mgpA* may be more active under high Mg²^+^ conditions (e.g., 10 mM). The lysates had no effect on growth when the medium contained 1 mM MgCl₂ (Fig. S9C through F), consistent with the growth enhancement resulting from the Mg²^+^ in the lysates.

These results suggest that lysates from the ∆*mgpA* ∆*corC* strain contain higher levels of Mg²^+^ than wild-type lysates. The ∆*corC* strain appeared to retain slightly more Mg²^+^ than wild type when cells are grown in 1 mM MgCl₂, while the ∆*mgpA* strain seemed to contain more Mg²^+^ when grown in 10 mM MgCl₂. These observations support our hypothesis that the CorC-regulated transporter(s) and MgpA transport excess Mg²^+^ to regulate intracellular cation levels.

### The *corC* and *mgpA* genes are induced in response to high Mg^2+^ and pH

**Fig 10:**
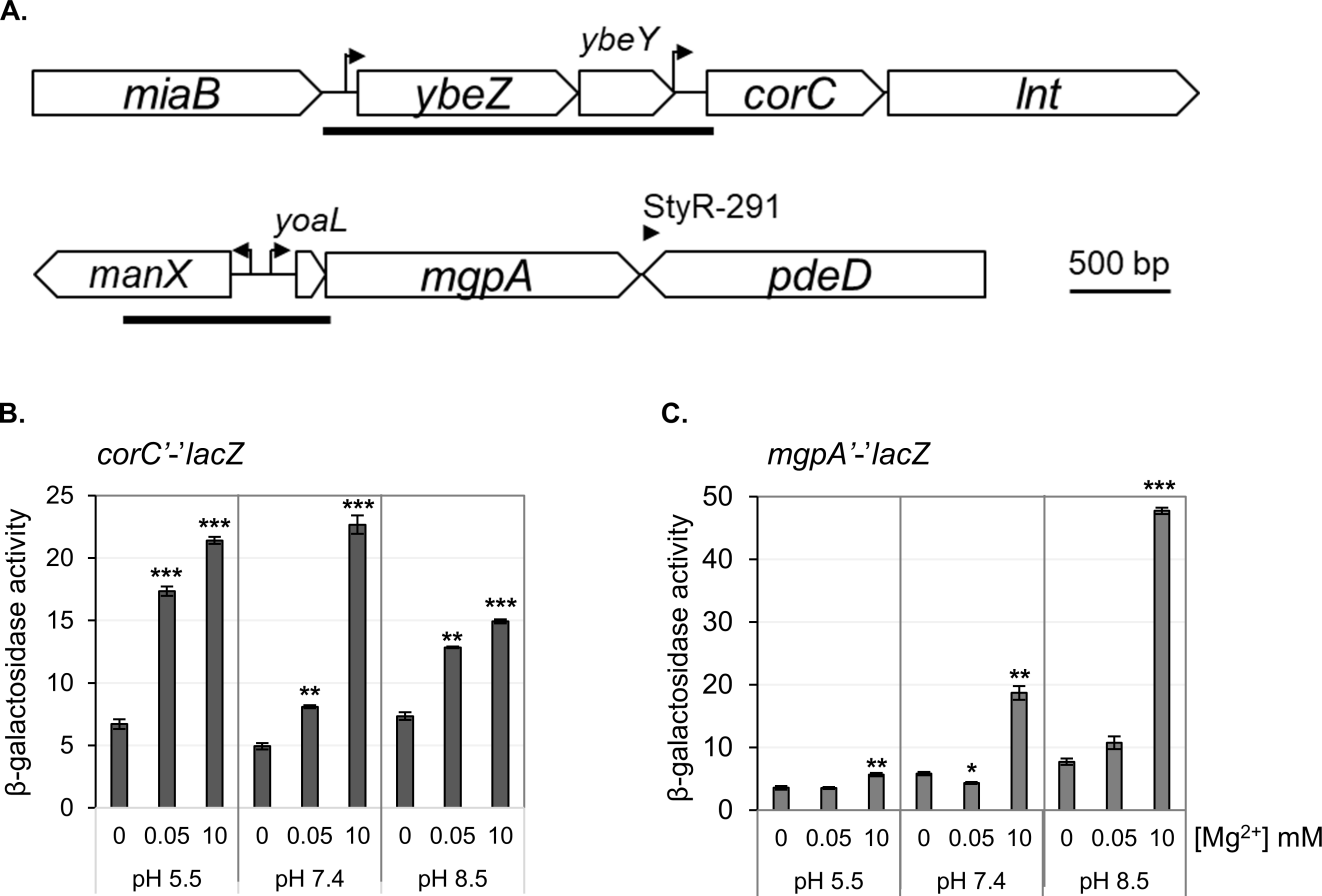
Expression of *mgpA* and *corC* is induced in high Mg^2+^ conditions. (**A**) The *corC* and *mgpA* loci. Dark bars indicate the sequences cloned upstream and in-frame with *lacZ*. The (**B**)* corC’-’lacZ* and (**C**)* mgpA’-’lacZ* fusion strains were pre-grown to mid-exponential phase in N-minimal medium pH 7.4 with 1 mM MgCl_2_ at 37°C, washed, diluted in N-minimal medium pH 5.5, pH 7.4, and pH 8.5 with or without the indicated amount of MgCl_2_, and incubated at 37°C for 24 hours. β-galactosidase activity was measured as described in Materials and Methods section. Values are mean ± SD, *n* = 6. Paired *t-*test (*P* < 0.05*, 0.005**, 0.0005***) versus 0 mM Mg^2+^ at the same pH. Strains used: JS2734 and JS2735.

We examined the expression of *corC* and *mgpA* in *Salmonella* using *lacZ* translational fusions. These constructs (Fig. 10A), containing the regions upstream of *corC* or *mgpA* and part of the open reading frames fused in frame to *lacZ*, were inserted at the λ *att* site in the chromosome. Thus, the chromosomal *corC* and *mgpA* loci were not disrupted. We measured expression after growth in different pH conditions (acidic [pH 5.5], neutral [pH 7.4], or alkaline [pH 8.5]) and in varying Mg^2+^ concentrations (0, 0.05, or 10 mM MgCl_2_). Expression of *corC* increased under high Mg^2+^ (10 mM) conditions, regardless of the pH of the medium (Fig. 10B). Expression of *mgpA* was elevated in high Mg^2+^ (10 mM) when the pH was neutral or alkaline (Fig. 10C). Thus, the expression of both CorC and MgpA is induced by high Mg²^+^ concentrations, consistent with a proposed role in combating excess-Mg²^+^ stress, with phenotypes exacerbated at high pH.

### MgpA and CorC are required for acute systemic infection

We previously demonstrated that the polyamine efflux transporter PaeA is required for full virulence in mice (7). Interestingly, the virulence attenuation of the *paeA* mutant varied depending on the mouse strain: C3H mice, with functional NRAMP1 (SLC11A1), versus BALB/c mice, which have a mutated NRAMP1 (34). NRAMP1 is a divalent cation transporter in the phagosomal membrane (27) that restricts *Salmonella* by reducing Mg²^+^ availability in phagosomes (26). The ∆*paeA* strain showed reduced virulence in both strains of mice, but the effect was stronger in the C3H background, supposedly due to lower Mg²^+^ levels in the phagosomes (7). To examine the effect of deleting the *mgpA* and *corC* genes in acute systemic infection *in vivo*, we performed competition assays after intraperitoneal infection (IP) in C3H mice or BALB/c mice. As shown in Table 1, the ∆*corC* ∆*mgpA* strain showed attenuated virulence only in BALB/c mice. In C3H mice, the ∆*mgpA* ∆*corC* strain was equally virulent to the wild type. Note that in the repeat of these experiments (second set of five mice each), the C3H and BALB/c mice were injected with the same inoculum. These results suggest that MgpA and CorC are important for virulence when the Mg²^+^ levels in the phagosome are only moderately limited. Collectively, we found that MgpA and CorC are required for acute infection in the BALB/c mouse model. Conversely, PaeA is preferentially required for acute infection in the C3H mouse model. This indicates that the functions of MgpA and CorC are distinct from that of PaeA during *Salmonella* pathogenesis.

**TABLE 1:** Competition assays with the ∆*mgpA* ∆*corC* mutant

Strain A^*a*^	Strain B^*a*^	Mouse strain	No. of mice	Organ	Avg CFU recovered per organ	Median CI^*b*^	*P* value^*c*^	Fold change^*d*^
Δ*mgpA* Δ*corC*	WT	C3H	10	Spleen	5.1 × 10^5^	0.82	NS	~1
				Liver	3.6 × 10^6^	1.01	NS	~1
		BALB/c	10	Spleen	7.3 × 10^6^	0.18	0.0006	5.6
				Liver	2.9 × 10^7^	0.10	0.0006	10.5

^*a*^ Strains used were 14028 and JS2694.

^*b*^ Bacteria were recovered from the spleen and liver after intraperitoneal (i.p.) competition assays. The competitive index (CI) was calculated as (percent strain A recovered/percent strain B recovered)/(percent strain A inoculated/percent strain B inoculated).

^*c*^ A nonparametric test, the Mann–Whitney U test, was used to compare the CI values to the inocula. NS, not significant.

^*d*^ The fold attenuation is 1 /CI, reflecting the level of attenuation of strain A relative to strain B.

### MgpA and CorC are required for egg white tolerance

**Fig 11:**
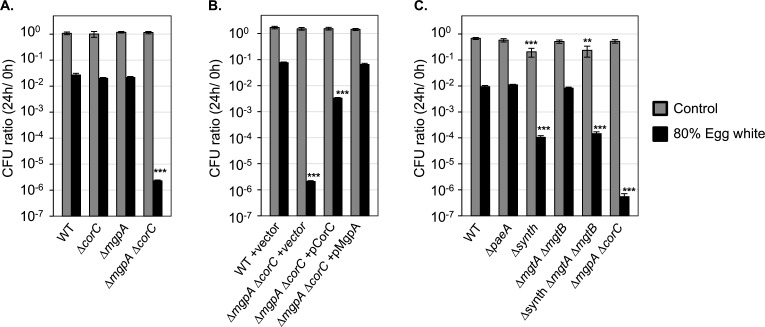
The ∆*mgpA* ∆*corC* strain is sensitive to egg white (A–C). The indicated strains were pre-grown to mid-exponential phase in N-minimal medium (pH 7.4) with 10 mM MgCl_2_ at 37°C, washed, diluted into 80% egg white solution or PBS (*t *= 0 h), and incubated at 37°C. CFUs were determined at 0 hour and 24 hours. CFU ratio (24 h/0 h) values are mean ± SD, *n* = 6. Unpaired t-test (*P* < 0.05*, 0.005**, 0.0005***) versus WT same treatment. Strains used: 14028, JS2728, JS2729, JS2730, JS2699, JS2731, JS2732, JS2733, JS2430, JS2560, JS2562, JS2563, and JS2694.

To further investigate the role of CorC and MgpA in *Salmonella* pathogenesis, we next focused on egg white tolerance. It was reported that loss of MgpA (YoaE) confers sensitivity to egg white in *S. Enteritidis*, the major serovar linked to human salmonellosis from contaminated eggs (35). We tested egg white tolerance in wild-type, ∆*mgpA*, ∆*corC*, and ∆*mgpA* ∆*corC* strains of* S. Typhimurium*. As shown in Fig. 11A, deletion of both *mgpA* and *corC* conferred increased sensitivity to egg white, whereas the individual deletions had no effect. The introduction of plasmids encoding either CorC or MgpA suppressed this egg white sensitivity (Fig. 11B). These phenotypes differ from those seen in *S. Enteritidis*, in which the single deletion of the *mgpA* gene resulted in sensitivity to egg white (35). These results indicate that *Salmonella* Typhimurium requires either MgpA or CorC to protect from egg white, although the mechanism is unknown.

To further understand the relationship between excess-cation stress and egg white stress, we examined whether other genes involved in reducing excess-cation stress are involved in egg white tolerance. As shown in Fig. 11C, mutants lacking PaeA, responsible for polyamine efflux, or MgtA and MgtB, the inducible Mg^2+^ transporters, showed similar viability as the wild type upon egg white exposure, suggesting that these transporters are not involved in egg white tolerance. Interestingly, the ∆synth mutant, lacking genes for polyamine synthesis (7, 14), exhibited sensitivity to egg white, although the degree of viability loss in the ∆synth mutant was smaller than in the ∆*mgpA* ∆*corC* strain. These results suggest that polyamine synthesis is required for survival in egg white, while polyamine efflux is not necessary. Altogether, we found that MgpA and CorC are required for egg white tolerance, while PaeA has no apparent role during egg white tolerance. These findings show that the functions of MgpA and CorC are distinct from that of PaeA during egg white exposure.

## DISCUSSION

Maintaining proper cation homeostasis in response to changes in the environment is essential for all living organisms. Previous studies reveal that, upon Mg^2+^ starvation, *Salmonella* induces the Mg^2+^ transporters MgtA and MgtB (9–13) and stimulates the synthesis of polyamines: cadaverine, putrescine, and spermidine (7, 8). Although the specific roles of polyamines are not fully understood, we propose that they act as simple cations that can substitute for Mg^2+^ to neutralize negative charges within cells. Once the Mg^2+^ concentration is re-established, the inner membrane transporter PaeA is required to efflux putrescine and cadaverine from the cell. Otherwise, when *Salmonella* enters stationary phase, the cell experiences what we term “excess-cation stress” (7) (Fig. 1). In the ∆*paeA* strain, polyamine accumulation occurs, leading to a loss of viability after Mg^2+^ starvation in stationary phase. This loss of viability can be suppressed by deleting the polyamine synthesis genes or the inducible Mg^2+^ transporter genes, showing that it is the combined concentration of polyamines and Mg that is lethal in stationary phase (7).

PaeA contains a CorC domain at its C-terminus (Fig. 2). Although some CorC domain-containing proteins have been implicated in metal ion homeostasis, the exact function of this domain is not understood. Here we have shown that two additional CorC domain-containing proteins in *Salmonella*, CorC and MgpA (YoaE), play a role in protecting against excess-cation stress in stationary phase. After growth in Mg^2+^-limiting medium, a *corC* deletion mutant loses viability in stationary phase. Deletion of *mgpA* alone has no effect but confers a synergistic phenotype in the *corC* background; the double mutant suffers a precipitous loss of viability in stationary phase after Mg^2+^ starvation (Fig. 3B). These proteins act independently of PaeA. Deletion of *paeA* has an apparent additive effect in the *corC* and *corC mgpA* backgrounds, and deletion of *mgpA* confers no additional phenotype in the *paeA* mutant (Fig. 3A). Loss of viability in the *corC mgpA* double mutant is completely suppressed by loss of the inducible Mg^2+^ transporters, MgtA and MgtB, and partially suppressed by deletion of the polyamine synthesis genes (Fig. 4). Thus, CorC and MgpA carry out some seemingly redundant function that is distinct from PaeA, yet all act to prevent excess-cation stress in stationary phase (Fig. 1).

The concentrations of both Mg^2+^ and polyamines in *Salmonella* range from 5 to 40 mM and are inversely and coordinately regulated by unknown mechanisms (7). Both Mg^2+^ and polyamines have high affinities for phosphate-containing compounds and should compete for binding. For example, putrescine has an ~10-fold higher affinity for ATP than does Mg^2+^ (36, 37). It is also known that putrescine can functionally replace ~80% of the Mg^2+^ in the ribosome (38). The cause of lethality under our conditions is not clear. Simplistically, lethality occurs when the combined concentrations of Mg^2+^ and polyamines are too high. However, it seems more likely that competition for binding to some specific compound in the cell is interfering with an essential function(s). Thus, it is the balance of cations and binding sites that is critical, rather than absolute levels. Our data are all consistent with PaeA exporting putrescine and cadaverine (7, 14). A previous study using intact cells implicates CorC in Mg²^+^ efflux (17). Given the apparent redundancy between MgpA and CorC, as evidenced by the synergistic phenotypes, we presume that MgpA is also an ion transporter. Expression of both is induced in high Mg²^+^. Our bioassay results show that the ∆*corC* ∆*mgpA* mutant grown in high Mg²^+^ accumulates higher intracellular Mg²^+^ levels compared to wild type (Fig. 9). The simplest model is that CorC and MgpA are responsible for efflux of excess Mg²^+^. However, we cannot rule out control or some other ion that is competing with, or for, Mg^2+^ and polyamines.

Further evidence shows that CorC/MgpA are functionally distinct from PaeA. The ∆*paeA* mutant, grown to stationary phase and suspended in high pH buffer, is sensitive to added cadaverine or putrescine (14). The polyamines are deprotonated at high pH, passively enter the cells, and, in the absence of PaeA, build to lethal concentrations. This lethality can be moderated by pre-growing the cells in low Mg^2+^ (7). Loss of CorC and MgpA rather partially suppresses this sensitivity to polyamines (Fig. 7). In this situation, where polyamines continue to enter the cytoplasm, the functions of CorC and MgpA negatively affect survival. One possibility is that the increased levels of polyamines compete with Mg^2+^, and this liberated Mg^2+^ results in a shift in ion balance and loss of viability. In trying to compensate for this stress by exporting excess Mg²^+^, CorC and MgpA are contributing to cation imbalance in this unusual situation.

Loss of CorC confers resistance to a high concentration of Mn^2+^ or Co^2+^ in the medium. MgpA has a more minor role in this phenotype (Fig. 6). Loss of CorA also confers resistance to Mn^2+^ or Co^2+^, as previously shown (17). Deletion of *corC* in the *corA* background confers additional protection. CorA also did not affect lethality in stationary phase after Mg^2+^ starvation (Fig. 4). Thus, CorC acts independently of CorA. Indeed, none of the previously identified cobalt-resistance loci, *corA*, *corB*, or *corD* (17), affect lethality in stationary phase after Mg^2+^ starvation (Fig. S4). When extracellular Mn^2+^ or Co^2+^ enter the cytoplasm, the functions of CorC and MgpA negatively impact cell survival. These results suggest that, analogous to sensitivity to external polyamines, when Mn^2+^ or Co^2+^ enter the cytoplasm, primarily through CorA-dependent mechanisms (39, 40), the increased levels of Mn^2+^ or Co^2+^ compete with and liberate Mg^2+^. CorC and MgpA export this “excess” free Mg²^+^, resulting in increased toxicity and a loss of viability.

The ∆*corC* ∆*mgpA* mutant also behaves very differently than the ∆*paeA* mutant in two virulence models. The ∆*corC* ∆*mgpA* mutant is also attenuated in competition assays in BALB/c mice, but, strikingly, there is no phenotype in C3H mice (Table 1). In contrast, the ∆*paeA* mutant shows a stronger attenuation in C3H mice, although there is still some phenotype in the BALB/c background (7). NRAMP1 is a divalent cation transporter in the phagosomal membrane that restricts *Salmonella* by reducing Mg²^+^ availability in phagosomes (26, 27). C3H mice have a functional NRAMP1 (SLC11A1), while BALB/c mice have a mutated NRAMP1 (34). It is clear that the macrophage phagosome in BALB/c is low in Mg²^+^ because the PhoPQ operon is strongly induced (7, 41). Presumably, the Mg²^+^ concentration is higher in the BALB/c phagosome than in the C3H phagosome, which could explain the increased sensitivity of the ∆*corC* ∆*mgpA* mutant. However, we cannot rule out other parameters in the phagosome that could differ between the mouse strains, affecting the overall environment and/or *Salmonella*’s regulatory response.

The ∆*corC* ∆*mgpA* double mutant is sensitive to egg white, whereas loss of PaeA has no apparent effect (Fig. 11). Huang et al. (35) demonstrated that the combination of high pH and a small peptide (smaller than 3 kDa) that is sensitive to proteinase K is necessary for egg white to kill the *mgpA* (*yoaE*) strain in *S. Enteritidis*. Our *in vitro* data show that the combination of high pH and high Mg²^+^ kills the ∆*corC* ∆*mgpA* double mutant (Fig. 8). Given that egg white contains 3.7–4.9  mM Mg²^+^ (42), it is possible that the double mutant is killed by the combined effects of high pH and elevated Mg²^+^ in egg white. However, other factors, such as ovotransferrin, which binds divalent cations (42), may also be involved. Further investigation into the molecular mechanisms of MgpA and CorC will deepen our understanding of how egg white kills *Salmonella*.

The pH of the media affects the phenotypes. The *corC mgpA* mutant loses viability after growth in Mg^2+^ starvation conditions in pH 7.4 or 8.5 medium (Fig. 3B and 5B). However, this phenotype is dramatically reduced when the pH of the medium is 5.5 (Fig. 5A). High pH also enhances sensitivity to high Mg²^+^ in the medium; the *corC mgpA* mutant cannot grow in pH 8.5 medium when the Mg^2+^ concentration is ≥10 mM, whereas the mutant grows fine in pH 7.5 medium, even at 100 mM Mg^2+^ (Fig. 8). There are several possibilities for these phenotypic differences at acidic, neutral, and alkaline pH. One possibility is that there are one or more redundant systems that reduce excess-cation stress in acidic environments. For example, PaeA and the cadaverine exporter CadB are redundant in acidic conditions (7). Another possibility is that the stress itself is reduced or alleviated in acidic environments, such that CorC and MgpA are no longer required. High pH in the medium can also significantly affect the activity of bacterial transporters, particularly those that rely on proton-motive force (43). Thus, under high pH conditions, bacteria might be particularly sensitive to high- or low-cation concentrations in the medium. However, these phenotypes are seemingly contradictory to the mouse phenotypes, given that the macrophage phagosome is low pH. Further investigation is necessary to address these questions.

Consistent with their roles in protecting against excess-cation stress, we demonstrated that *mgpA* expression is elevated in high Mg^2+^ (10 mM) when the pH is neutral or alkaline, whereas *corC* expression increases under high Mg^2+^ (10 mM) conditions, regardless of the pH of medium (Fig. 10). These results support the model that CorC and MgpA are involved in mitigating high-Mg^2+^ stress. The regulatory mechanisms governing *mgpA* and *corC* expression remain unclear. Chadani et al. showed that the translation of the N-terminal region of CorC (YbeX) contains an intrinsic ribosome destabilization (IRD) sequence (44). This IRD sequence destabilizes the translating ribosomal complex, leading to premature termination of translation under conditions in which ribosome function is reduced. Low-Mg^2+^ stress is a well-known example of ribosome-destabilizing conditions. For example, under such stress, the translation of MgtL, a leader peptide for the inducible magnesium transporter MgtA, stalls due to an IRD mechanism, resulting in the upregulation of *mgtA* expression (45, 46). Consequently, CorC expression is likely upregulated when cytoplasmic Mg^2+^ levels exceed a certain threshold. As for *mgpA* regulation, a leader peptide, YoaL, overlaps with the upstream region of the *mgpA* gene (Fig. 10A (47). Replacing the start codon of YoaL with a stop codon leads to decreased MgpA translation (48). This suggests that YoaL might play a role in upregulating *mgpA* expression in response to high Mg^2+^ levels. Further research is required to test this hypothesis and to identify the signals that influence this leader peptide-mediated regulation of *mgpA* expression.

Based on the presence of a transmembrane TerC domain in the N-terminus of MgpA (Fig. 2), which is similar to a protein in *Bacillus subtilis* known to be involved in Mn transport (49), MgpA appears to function directly as a cation transporter. In contrast, CorC, unlike other CorC domain-containing proteins, is cytoplasmic and contains only a CBS-pair domain and the CorC domain. Studies have shown that inactivation of the *corC* gene results in decreased Mg^2+^ efflux activity under high Mg^2+^ conditions. When *corC* is inactivated along with *corB* and *corD* (*apaG*) genes, Mg^2+^ efflux activity is completely lost (17). These data would suggest that CorC regulates a protein responsible for Mg^2+^ efflux activity. However, the protein that CorC regulates does not appear to be CorA, CorB, CorD, or any other CorC-domain-containing proteins, including MgpA, because deletion of the *corC* gene in strains lacking these proteins still confers phenotypes (Fig. 4 and S4). Therefore, we hypothesize that CorC mitigates excess-cation stress, in response to Mg^2+^ levels, by regulating an uncharacterized transporter. This also leads to the general conclusion that the CorC domain, likely in conjunction with the CBS domain, is regulatory, perhaps sensing levels of cytoplasmic cations. Indeed, a crystal structure of the CorB CBS pair from the thermophilic archaeon *Methanoculleus thermophilus* shows that dimerization is dependent on binding of ATP-Mg^2+^, with the Mg^2+^ ion coordinated between the dimers (16), potentially providing a mechanism for sensing relative Mg^2+^ levels.

Mg^2+^ is critical for ribosome assembly, function, and stability (4, 6). It is also required for stability of the outer membrane, neutralizing the negative charges in outer membrane lipopolysaccharide (LPS) (5, 50). Indeed, low Mg^2+^ induces stress on outer membrane homeostasis and biogenesis (51). Interestingly, the *corC* gene is located in an operon downstream of *ybeZ* and *ybeY*, and upstream of *lnt*. In addition to the promoter upstream of *ybeZ*, there is a start site of transcription between *ybeY* and *corC* (Fig. 10A) (22, 52). YbeY is an endoribonuclease with pleiotropic effects on ribosome maturation and function (53). The upstream YbeZ is a putative RNA helicase that is also proposed to participate in ribosome maturation (54). Lnt (CutE), encoded downstream of CorC, is an essential protein required for the final acylation of the N-terminal Cys residue of processed outer membrane lipoproteins (55), including the major lipoprotein Lpp, which tethers the outer membrane to the peptidoglycan (56).

Sarigul et al. (23) explored the role of CorC in ribosome metabolism in *E. coli*. They demonstrated that a *corC* deletion mutant exhibited an extended lag phase coming out of stationary phase, particularly when cells were grown at elevated temperatures and under low Mg^2+^ conditions. The *corC* mutant also displayed increased sensitivity to several ribosome-targeting antibiotics. The accumulation of 17S pre-rRNA, a precursor of 16S rRNA, and partial degradation intermediates of 16S rRNA was noted in the *corC* deletion strain upon entering stationary phase. Importantly, the growth phenotypes were suppressed by adding additional Mg^2+^ to the growth medium. Orelle et al. (57) constructed an *E. coli* “Ribo-T” strain that expresses an engineered hybrid rRNA composed of both 16S and 23S rRNA, such that the ribosome subunits are tethered and inseparable. A strain making only these engineered ribosomes grew slowly. Selecting for a faster-growing mutant yielded a nonsense mutation in *corC,* together with a missense mutation in *rpsA*. Thus, loss of CorC affects ribosome stability and/or function in a Mg^2+^-dependent manner. Overall, the coordinate regulation of these various genes suggests that CorC has an important role in both Mg^2+^-dependent ribosome function and outer membrane stability. Further investigation into the biochemical function of CorC and MgpA is required to understand how these proteins reduce excess-cation stress and how regulation of cation levels affects overall cell physiology and survival in host tissues.

## MATERIALS AND METHODS

### Media

During construction of strains, bacteria were routinely grown at 37°C in Luria-Bertani (LB) medium containing 1% NaCl. Strains containing temperature-sensitive plasmids pCP20 and pKD46 were grown at 30°C. When necessary, antibiotics were used at the following concentrations: ampicillin, 50  µg/mL; chloramphenicol, 10  µg/mL; kanamycin, 50  µg/mL; and apramycin, 50  µg/mL. N-minimal medium (9, 58) contains 5 mM KCl, 7.5 mM (NH_4_)_2_SO_4_, 0.5 mM K_2_SO_4_, 1 mM KH_2_PO_4_, 0.1 M Tris, 15 mM glycerol, and 0.1% casamino acid. The pH was adjusted as indicated. MOPS medium (59), adjusted to pH 7.4 with KOH, contains 40 mM MOPS, 4 mM Tricine, 9.5 mM NH₄Cl, 0.276 mM K₂SO₄, 50 mM NaCl, and 0.1% casamino acids. When indicated, 1.32 mM K₂HPO₄ and 10 mM MgCl₂ were added. We did not add the trace metals listed in the original recipe (59) to minimize the effects of metal addition.

### Bacterial strains and plasmids

Strains are described in Table S1, while plasmids are listed in Table S2. All *Salmonella* strains used are derivatives of *Salmonella enterica* serovar Typhimurium strain 14028. Deletions with concomitant insertion of antibiotic resistance cassettes were constructed using λ Red-mediated recombination as previously described (60, 61), with the indicated endpoints (Table S1). Deletions were verified by PCR analysis, and then transduced into the appropriate backgrounds using phage P22 HT105/1 int-201 (62). All plasmids were passaged through a restriction-minus, modification-plus, Pi^+^ *Salmonella* strain (JS198) (61) prior to transformation into *Salmonella* strains. Antibiotic resistance cassettes were removed using the pCP20 plasmid. The resulting deletions were confirmed by PCR.

The translational out-of-locus *corC’-‘lacZ* and *mgpA’-‘lacZ* translational fusions were constructed by introducing the regulatory regions of *corC* and *mgpA*, along with the first or twentieth amino acids of the ORFs, respectively, amplified by PCR using primers listed in Table S3, upstream and in-frame with the promoterless *lacZ* in the pDX1 vector via Gibson assembly (NEBuilder HiFi DNA Assembly)(Fig. 10). The resulting product was transformed into DH5α λ*pir*, and the plasmid was confirmed by DNA sequencing. The plasmids, pDX1-*corC-lacZ* and pDX1-*mgpA-lacZ*, were integrated into the λ-attachment site in the *Salmonella* chromosome, as previously described (63). The primers used and the endpoints of the cloned fragments are indicated in Tables S2 and S3, respectively.

### Survival assay

Overnight cultures, grown in N-minimal medium (pH 7.4) supplemented with 10 mM MgCl_2_, were washed with 0.85% NaCl three times, diluted to an OD_600_ value of 0.0375 into 5 mL of N-minimal medium with the indicated amount of MgCl_2_, and incubated at 37°C on a roller drum in a 20 × 150 mm tube. Before and after incubation, serial dilutions of the cultures were plated on LB agar plates that were incubated overnight at 37°C to determine CFU. For the growth curves, the OD_600_ of 250 µL of culture was measured in a BioTek ELx808 Absorbance Reader at the indicated time points. When necessary, the cultures were diluted in the original growth medium to accurately measure the OD_600_ value.

### Mn^2+^ and Co^2+^ sensitivity assay

Overnight cultures, grown in modified MOPS medium (pH 7.4) supplemented with 15 mM glycerol, 1.32 mM K_2_HPO_4_, and 10 mM MgCl₂ were washed three times with 0.85% NaCl, diluted to an OD_600_ value of 0.05 into 10 mL of modified MOPS medium (pH 7.4) supplemented with 15 mM glycerol and with or without 1 mM MnCl_2_ or 0.2 mM CoCl_2_ in 125 mL baffled flask, and incubated at 37°C for 9 hours. Before and after incubation, serial dilutions of the cultures were plated on LB agar plates that were incubated overnight at 37°C to determine CFU.

### Polyamine sensitivity assay

Polyamine stocks were prepared at 500 mM in 0.85% NaCl with HEPES buffer (pH 8.5). Overnight cultures grown in N-minimal medium (pH 7.4) supplemented with 10 mM MgCl₂ were washed three times with 0.85% NaCl, then diluted 1:200 into 5 mL of N-minimal medium containing either 50 µM or 10 mM MgCl₂. These cultures were incubated at 37°C on a roller drum for 24 hours. Then, cells were washed three times, resuspended in the same volume of 0.85% NaCl with HEPES buffer (pH 8.5), and aliquoted into 1 mL portions in 13 × 100 mm test tubes. The indicated amounts of cadaverine, putrescine, or spermidine were added, and the cultures were incubated at 37°C on a roller drum. At 0 and 24 hours, serial dilutions of the cultures were plated on LB agar plates and incubated overnight at 37°C to determine the CFU.

### High pH growth assay

Overnight cultures grown in N-minimal medium (pH 7.4), supplemented with 15 mM glycerol and 1 mM MgCl₂, were diluted 800-fold into the same medium and grown for 4.5 hours. The cells were then washed three times with 0.85% NaCl, diluted to an OD₆₀₀ of 0.005 in 10 mL of N-minimal medium (pH 7.4 or 8.5) containing 15 mM glycerol and 1, 10, or 100 mM MgCl₂, and incubated at 37°C in a 125 mL baffled flask. The OD₆₀₀ of culture was measured using a BioTek ELx808 Absorbance Reader at the indicated time points. When necessary, cultures were diluted in the original growth medium to ensure accurate OD₆₀₀ measurements. To determine CFU, serial dilutions of the cultures were plated on LB agar plates and incubated overnight at 37°C.

### Bioassay for Mg^2+^

To prepare the lysates, overnight cultures grown in N-minimal medium (pH 7.4) supplemented with 15 mM glycerol and 10 mM MgCl₂ were diluted 750-fold into the same medium and grown for 4.5 hours. The cells were then washed three times with PBS, diluted to an OD₆₀₀ of 0.005 in 15 mL of N-minimal medium (pH 7.4) containing 15 mM glycerol and either 1 or 10 mM MgCl₂, and incubated at 37°C in a 125 mL baffled flask. After 24 hours, 10  µg/mL chloramphenicol was added to the culture and incubated for 5 min, followed by transfer of the flasks to ice-cold water for another 5 min. Both steps aimed to reduce cation efflux activity. Then, cells were collected by centrifugation (7,000 × *g*), washed once with PBS containing 100 mM EDTA, and then washed twice with Chelex-treated PBS. Cell pellets were resuspended in 350 µL of Chelex-treated PBS and sonicated. Debris was removed by centrifugation (12,000 × *g*). Total protein concentration was measured by a Bradford assay (Bio-Rad) and adjusted to 125 mg/mL with Chelex-treated PBS. The lysates were then boiled in a 98°C water bath for 15 min to denature proteins and release Mg2^+^.

To assess the growth-stimulating activity of the cell lysates, overnight cultures grown in N-minimal medium (pH 7.4) supplemented with 15 mM glycerol and 10 mM MgCl₂ were diluted 750-fold into the same medium and grown for 4.5 hours. Cells were then washed three times with PBS and diluted to an OD₆₀₀ of 0.005 in 250 µL of N-minimal medium (pH 7.4), either with or without the indicated concentrations of MgCl₂, or with or without 1 mM MgCl₂ supplemented with 34  µg/mL of each lysate. Cultures were incubated in a BioTek ELx808 Absorbance Reader with shaking for 15 hours, after which final yields (OD_600_) were measured using a BioTek ELx808 Absorbance Reader. When necessary, cultures were diluted in the original growth medium to ensure accurate OD₆₀₀ measurements.

### β-galactosidase assay

Overnight cultures grown in N-minimal medium (pH 7.4) supplemented with 10 mM MgCl₂ were diluted into the same medium and grown for 4 hours. The cells were then washed three times with 0.85% NaCl and diluted to an OD₆₀₀ of 0.025 in 25 mL of N-minimal medium (pH 5.5, pH 7.4, or pH 8.5) with the indicated amount of MgCl₂ in a 125 mL baffled flask, followed by incubation at 37°C. After 24 hours, 1 mL of culture was harvested, the cells were pelleted, resuspended in Z buffer, and assayed for β-galactosidase activity in a microtiter plate format, as previously described (64). The β-galactosidase activity units are defined as (μmol of ortho-nitrophenol formed per minute) × 10^6^ / (OD_600_ × mL of cell suspension) and are presented as the mean ± standard deviation with *n* = 6.

### Egg white sensitivity assay

Eggs were purchased from the Meat & Egg Sales Room in the Department of Animal Sciences in the University of Illinois at Urbana-Champaign and stored at 4°C until used (< one month). Egg white was collected from the eggs and mixed with a 4:1 vol of 0.85% NaCl. The mixture was homogenized on ice using a mortar and pestle, and debris was removed by centrifugation. The resulting solution was used as an 80% egg white solution. Note that these egg white solutions did not contain microbes capable of growing on an LB plate. Overnight *Salmonella* cultures grown in N-minimal medium (pH 7.4) supplemented with 10 mM MgCl₂ were diluted into the same medium and grown for 4 hours. The cells were then washed three times with 0.85% NaCl and diluted to an OD₆₀₀ of 0.02 in 1 mL of 80% egg white solution or 0.85% NaCl in 13 × 100 mm test tubes. The cultures were incubated at 37°C on a roller drum. At 0 and 24 hours, serial dilutions of the cultures were plated on LB agar plates that were incubated overnight at 37°C to determine the CFU.

### Animal assay

All of the animal work was reviewed and approved by the University of Illinois IACUC and was performed under the protocol number 21197. The competition experiments were performed using 5- to 6-week-old mice. The BALB/cAnNHsd and C3H/HeNHsd mice were purchased from Envigo. The *Salmonella* strains were grown overnight in LB medium, mixed 1:1, and diluted to a target inoculum of approximately 1,000 CFU in 200 µL sterile PBS. The mice were infected via the intraperitoneal route. Each inoculum was plated on LB medium to measure the total inoculum and was replica plated onto the appropriate selective medium in order to calculate the input ratio for each strain. After four days of infection for the BALB/cAnNHsd mice or five days of infection for the C3H/HeNHsd mice, the animals were sacrificed via CO_2_ asphyxiation and cervical dislocation, and their spleens and livers were removed and homogenized. Serial dilutions of the spleen and liver homogenates were plated on LB medium and incubated overnight. The resulting colonies were replica plated to the appropriate selective medium in order to calculate the output ratio for each competition. The competitive index (CI) was calculated as (percent strain A recovered/percent strain B recovered)/(percent strain A inoculated/percent strain B inoculated). The statistical comparisons of individual competitions were performed using a nonparametric Mann–Whitney test.

## SUPPLEMENTAL MATERIAL

Supplemental figures and tables mbio.02021-25-s0001.pdf Fig. S1–S9; Tables S1–S3.

ASM does not own the copyrights to Supplemental Material that may be linked to, or accessed through, an article. The authors have granted ASM a non-exclusive, world-wide license to publish the Supplemental Material files. Please contact the corresponding author directly for reuse.
