# Supplementary material for: The CorC proteins MgpA (YoaE) and CorC protect from excess-magnesium stress and are required for egg white tolerance and virulence in Salmonella
Source: mBio. 2025 Aug 18;16(9):e02021-25. doi: 10.1128/mbio.02021-25 (PMC12421838; doi:10.1128/mbio.02021-25)
Supplement: Supplemental figures and tables — Fig. S1–S9; Tables S1–S3. [file mbio.02021-25-s0001.pdf]

Supplemental Material

The CorC proteins MgpA (YoaE) and CorC protect from excess-magnesium stress and are required for egg white tolerance and virulence in *Salmonella*

Yumi Iwadate[1], and James M. Slauch[1*]

[1]Department of Microbiology, University of Illinois at Urbana-Champaign, Urbana, IL, 61801, USA.

*Correspondence: James M. Slauch
Department of Microbiology
University of Illinois
B103 CLSL, MC-110
601 S Goodwin Ave
Urbana, IL 61801
217-244-1956
slauch@illinois.edu

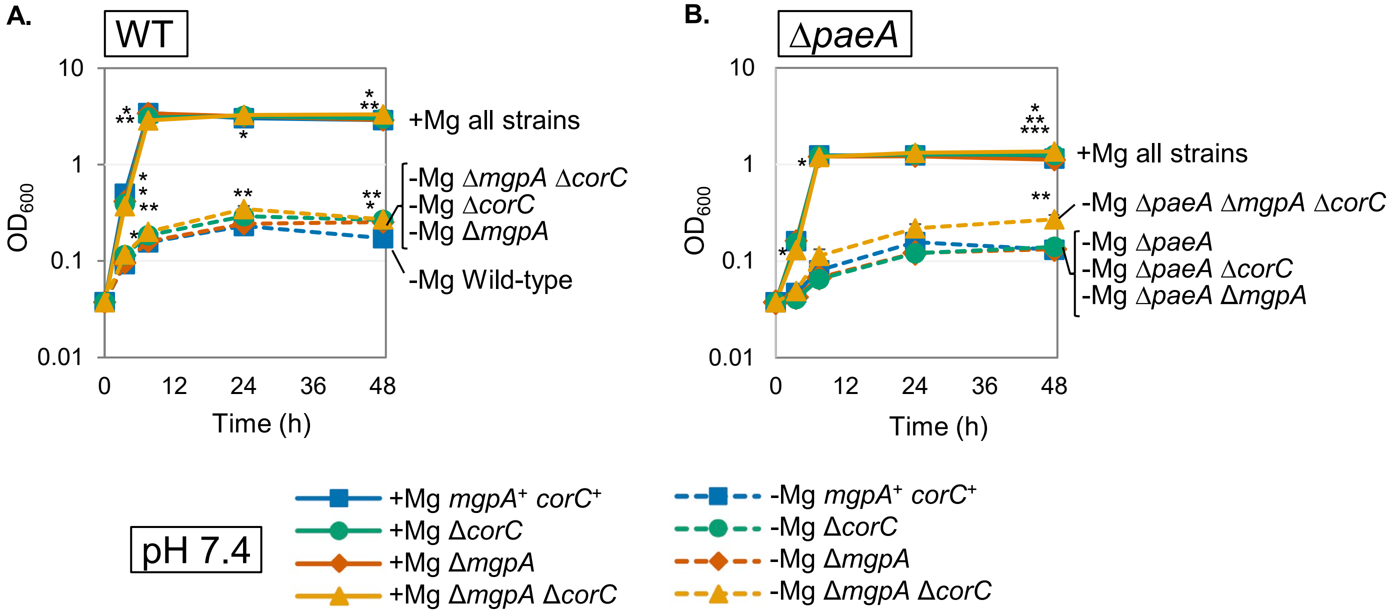

Fig. S1. OD_{600} of the indicated strains (same experiment as Fig.2) after incubation in Mg^{2+} starvation conditions . The indicated strains were grown overnight in N-minimal medium pH 7.4 with 10 mM $MgCl_2$, washed, diluted into N-minimal medium pH 7.4 with or without 10 mM $MgCl_2$ (t=0 h), and incubated at 37℃. OD_{600} was determined at the indicated time points in WT (A) and Δ*paeA* (B) backgrounds. Values are mean ± SD, n = 6. Unpaired t test ($p < 0.05$*, 0.005**, 0.0005***) vs *mgpA*⁺ *corC*⁺ parent strain at the same timepoint at the same time point and at the same Mg^{2+} concentration. Strains used: 14028, JS2692, JS2693, JS2694, JS2695, JS2696, JS2697, and JS2698.

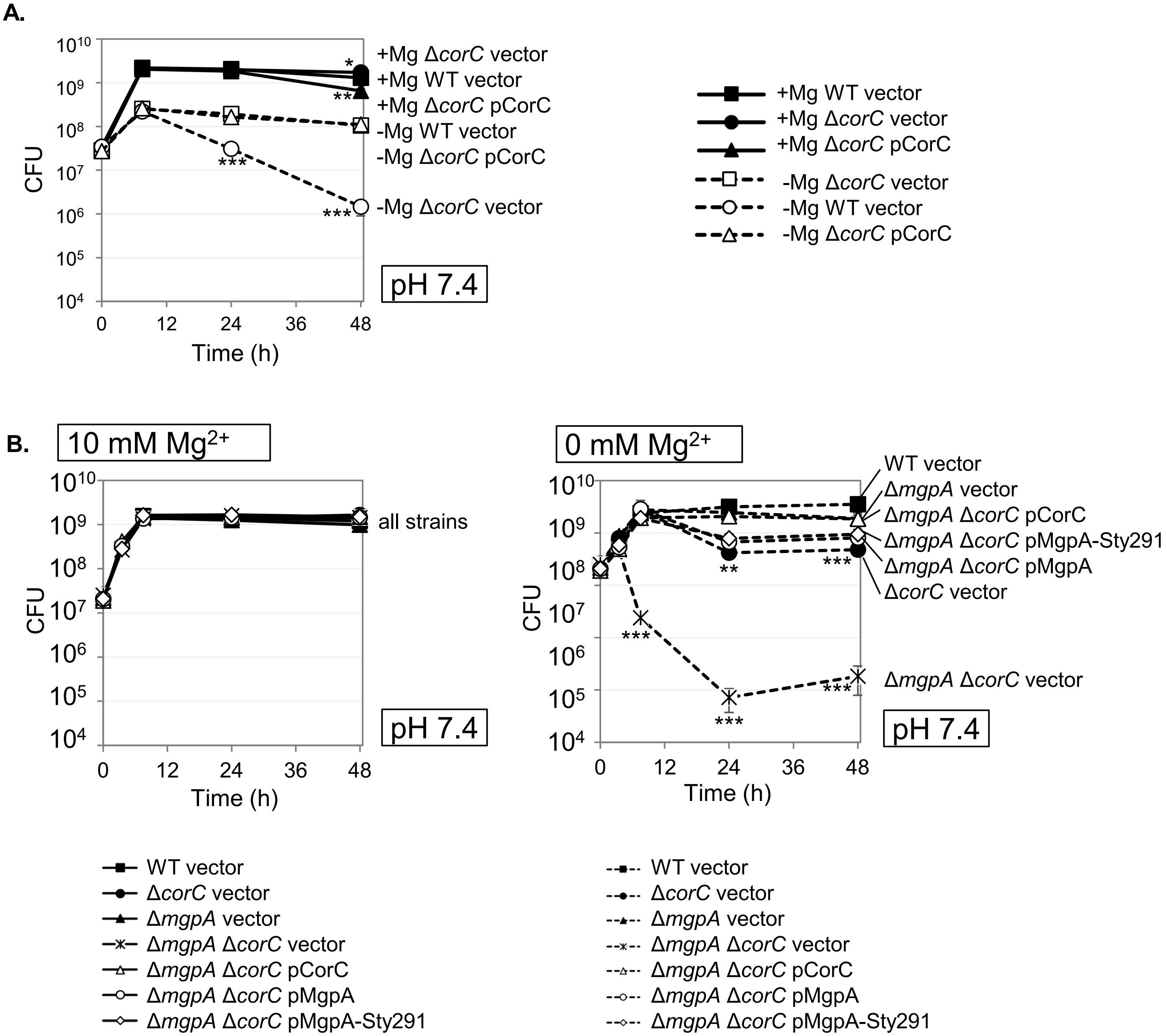

Fig. S2. Complementation tests in the indicated strains after incubation in Mg^{2+} starvation conditions. The strains containing pWKS30 plasmid (vector) or pCorC, pMgpA, or pMgpA-Sty291 plasmid as indicated were grown overnight in N-minimal medium pH 7.4 with 10 mM $MgCl_2$, washed, diluted into N-minimal medium pH 7.4 with or without 10 mM $MgCl_2$ (t=0 h), and incubated at 37℃. CFUs were determined at the indicated time points. Values are mean ± SD, n = 6. Unpaired t test (p < 0.05*, 0.005**, 0.0005***) vs WT vector strain at the same time point and at the same Mg^{2+} concentration. Strains used: JS2699, JS2700, JS2701, JS2702, JS2703, JS2704, JS2705, and JS2706

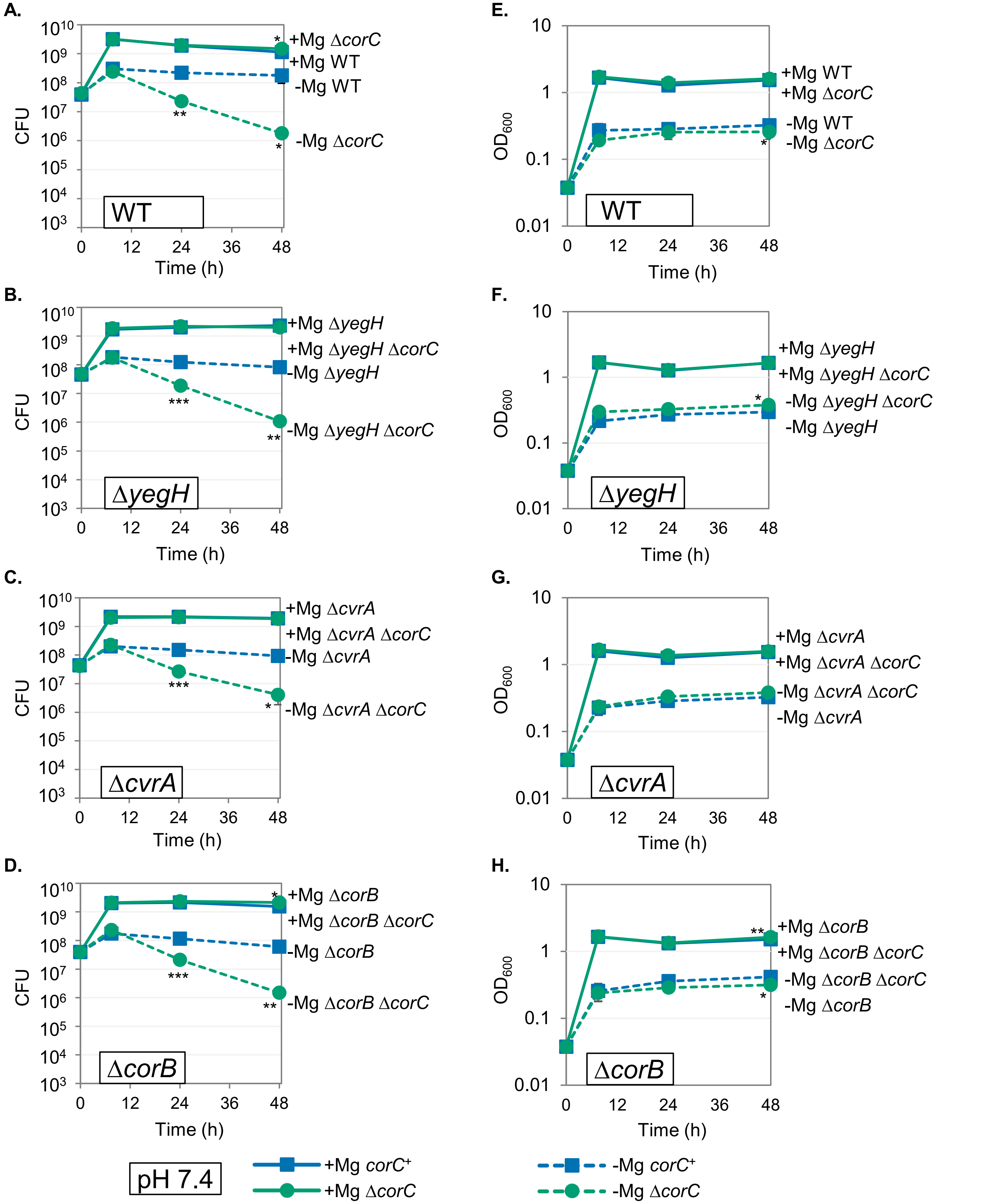

Fig. S3. YegH, CorB, and CvrA confer no phenotype in stationary phase after Mg^{2+} starvation. The indicated strains were grown overnight in N-minimal medium pH 7.4 with 10 mM $MgCl_2$, washed, diluted into N-minimal medium pH 7.4 with or without 10 mM $MgCl_2$ (t=0 h), and incubated at 37℃. CFUs and OD_{600} were determined at the indicated time points in WT (A,E), Δ*yegH* (B,F), Δ*cvrA* (C,G), and Δ*corB* (D,H) background. Values are mean ± SD, n = 6. Unpaired t test ($p < 0.05$*, 0.005**, 0.0005***) Δ*corC* vs corresponding *corC*+ parent strain at the same time point and at the same Mg^{2+} concentration. Strains used: 14028, JS2692, JS2707, JS2708, JS2709, JS2710, JS2711, and JS2712.

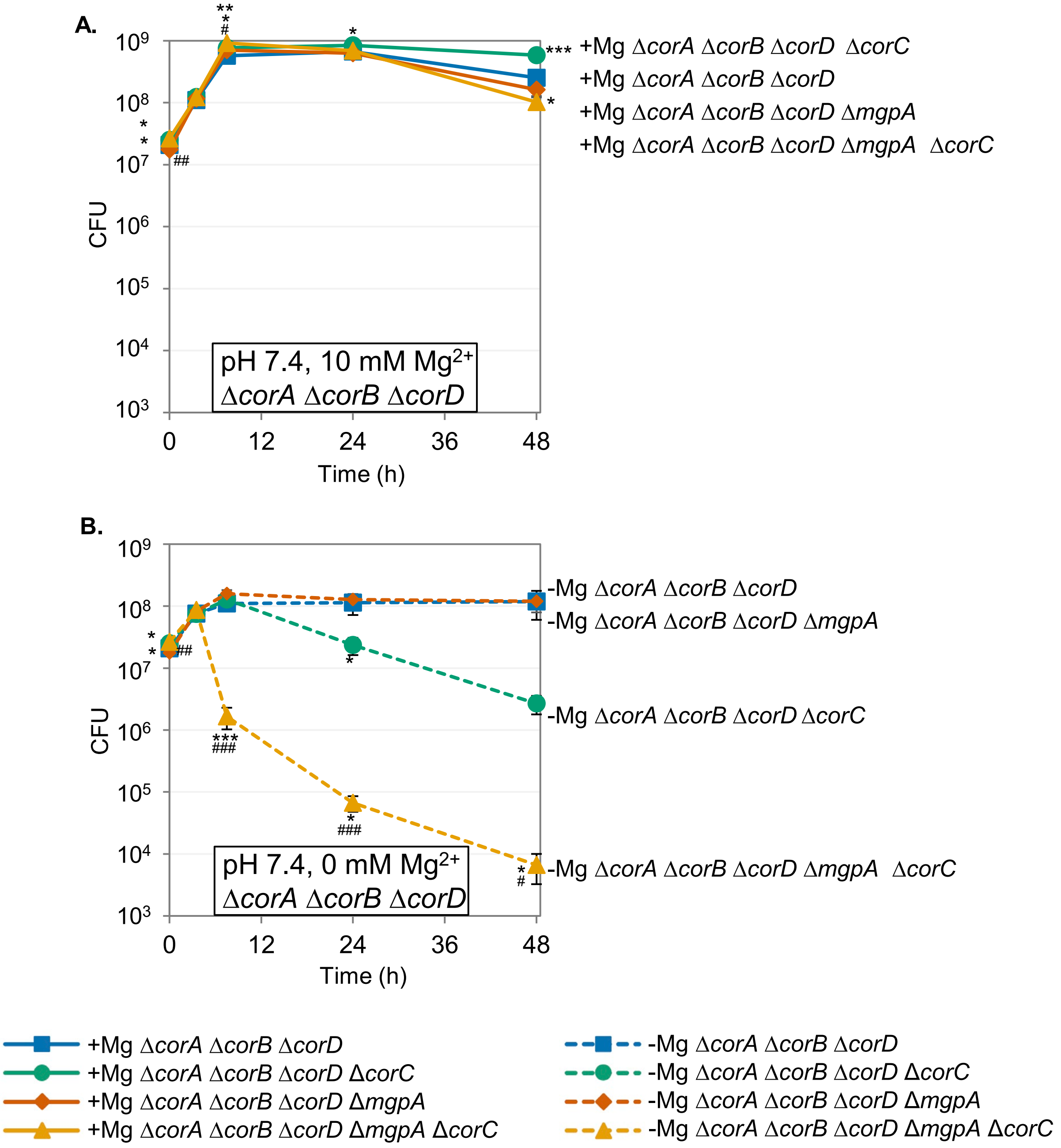

Fig. S4. Loss of CorA, CorB, and CorD does not affect survival of the ∆corC and ∆*corC* ∆*mgpA* strains in stationary phase after Mg^{2+} starvation. The indicated strains were grown overnight in N-minimal medium pH 7.4 with 10 mM $MgCl_2$, washed, diluted into N-minimal medium pH 7.4 (A) with or (B) without 10 mM $MgCl_2$, and incubated at 37℃. CFUs were determined at the indicated time points in the ∆*corA* ∆*corB* ∆*cor*D background. Values are mean ± SD, n = 6. Unpaired t test ($p < 0.05^{*}$, 0.005^{**}, 0.0005^{***}) versus the control strain at the same time point and at the same Mg^{2+} concentration and ($p < 0.05^{\#}$, $0.005^{\#\#}$, $0.0005^{\#\#\#}$) ∆*mgpA corC*+ versus ∆*mgpA* ∆*corC* at the same time point and at the same Mg^{2+} concentration. Strains used: 14028, JS2692, JS2693, JS2694, JS2713, JS2714, JS2715, JS2716, JS2560, JS2717, JS2718, JS2717, JS2720, JS2721, JS2722, and JS2723.

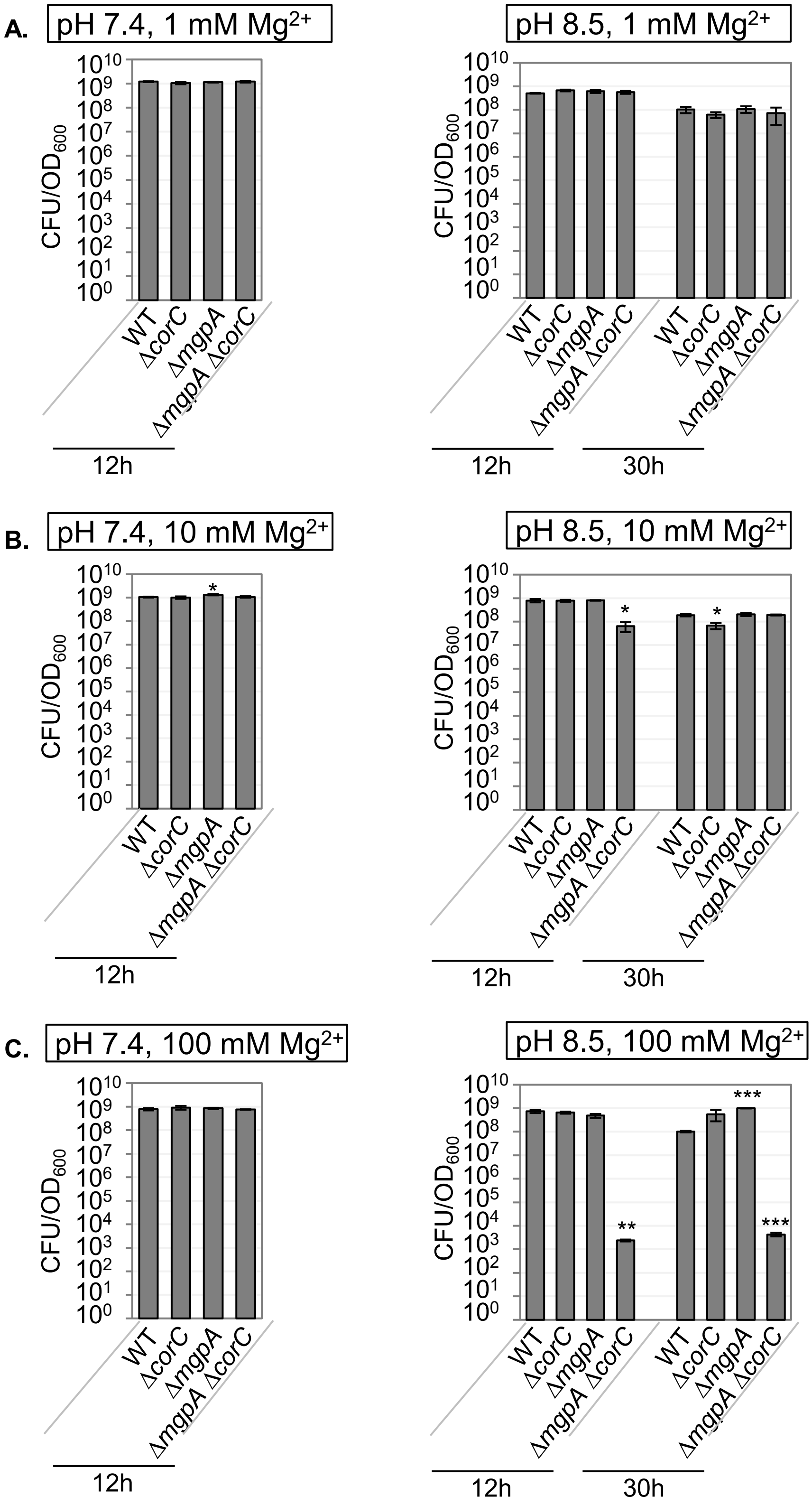

Fig. S5. The Δ*mgpA* Δ*corC* strain loses viability in the high Mg^{2+} and high pH medium. The indicated strains were pre-grown to mid-exponential phase in N-minimal medium pH 7.4 with 1 mM $MgCl_2$, washed, and diluted into N-minimal medium pH 7.4 or pH 8.5 with (A) 1 mM, (B) 10 mM, and (C) 100 mM $MgCl_2$ (t=0h), and incubated at 37℃. CFUs were determined at 12h and 30h. Corresponding OD_{600} measurements at the same timepoint from the same experiment are shown in Fig 8. CFUs/OD_{600} were calculated at 12h and 30h. CFU/OD_{600} values are mean ± SD, n = 3. Unpaired t test ($p < 0.05$*, 0.005**, 0.0005***) versus corresponding WT at the same timepoint. Strains used: 14028, JS2692, JS2693, and JS2694.

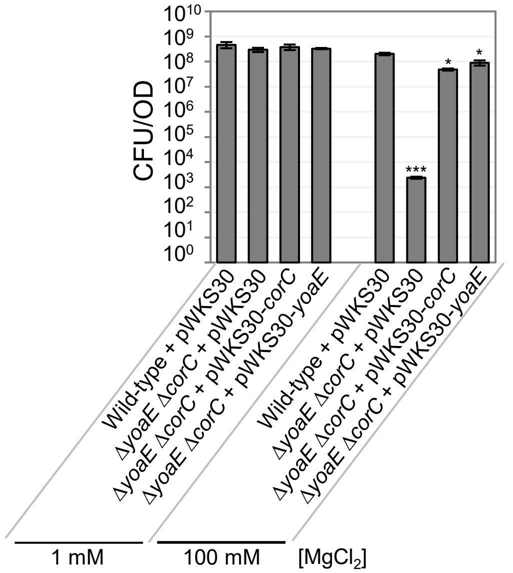

Fig S6. Complementation tests in the indicated strains under high Mg^{2+} conditions. The indicated strains were pre-grown to mid-exponential phase in N-minimal medium (pH 7.4) with 1 mM $MgCl_2$, washed, and diluted into N-minimal medium pH 8.5 with 1 and 100 mM of $MgCl_2$, and incubated at 37 ℃CFUs/OD_{600} were determined after 12 hours of incubation. Values are mean ± SD, n = 3. Unpaired *t* test vs corresponding wild-type strain with an empty vector (p < 0.05*, 0.005**, 0.0005***) at the same condition. Strains used: JS2699, JS2703, and JS2704.

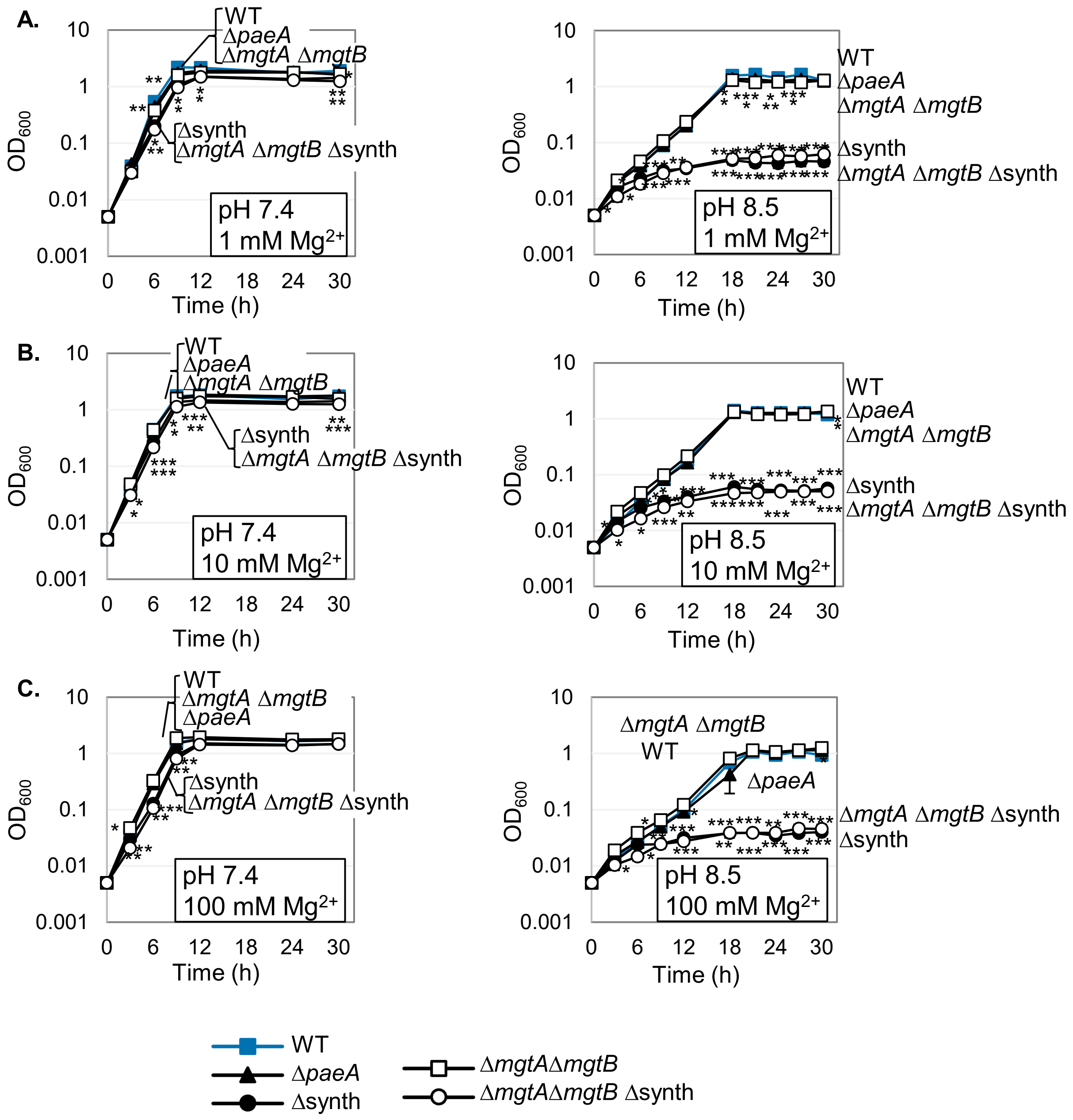

Fig. S7. pH affects growth under high Mg^{2+} conditions. The indicated strains were pre-grown to mid-exponential phase in N-minimal medium pH 7.4 with 1 mM $MgCl_2$, washed, and diluted into N-minimal medium pH 7.4 or pH 8.5 with (A) 1 mM, (B) 10 mM, and (C) 100 mM $MgCl_2$ (t=0h), and incubated at 37℃. OD_{600} was determined at the indicated timepoints. Values are mean ± SD, n = 3. Unpaired t test ($p < 0.05$*, 0.005**, 0.0005***) versus corresponding WT at the same timepoint. Strains used: 14028, JS2430, JS2560, JS2562, and JS2563.

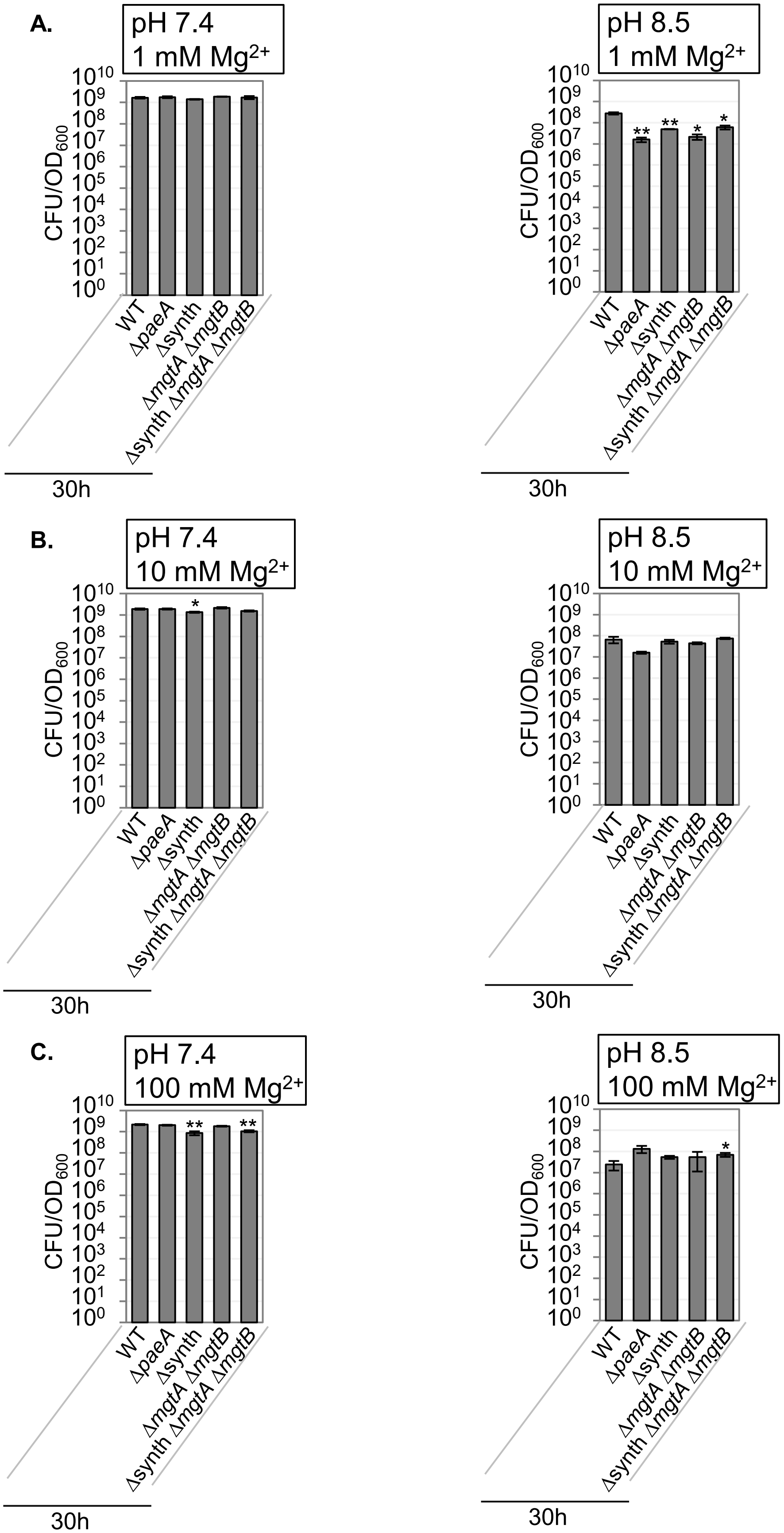

Fig. S8. The Δ*paeA* strain viability is not affected by the high Mg^{2+} and high pH. The indicated strains were pre-grown to mid-exponential phase in N-minimal medium pH 7.4 with 1 mM $MgCl_2$, washed, and diluted into N-minimal medium pH 7.4 or pH 8.5 with (A) 1 mM, (B) 10 mM, and (C) 100 mM $MgCl_2$ (t=0h), and incubated at 37℃. CFUs were determined at 30h. Corresponding OD_{600} measurements at the same timepoint from the same experiment are shown in Fig S6. CFUs/OD_{600} were calculated at 30h. CFU/OD_{600} values are mean ± SD, n = 3. Unpaired t test ($p < 0.05$*, 0.005**, 0.0005***) versus corresponding WT at the same timepoint. Strains used: 14028, JS2430, JS2560, JS2562, and JS2563.

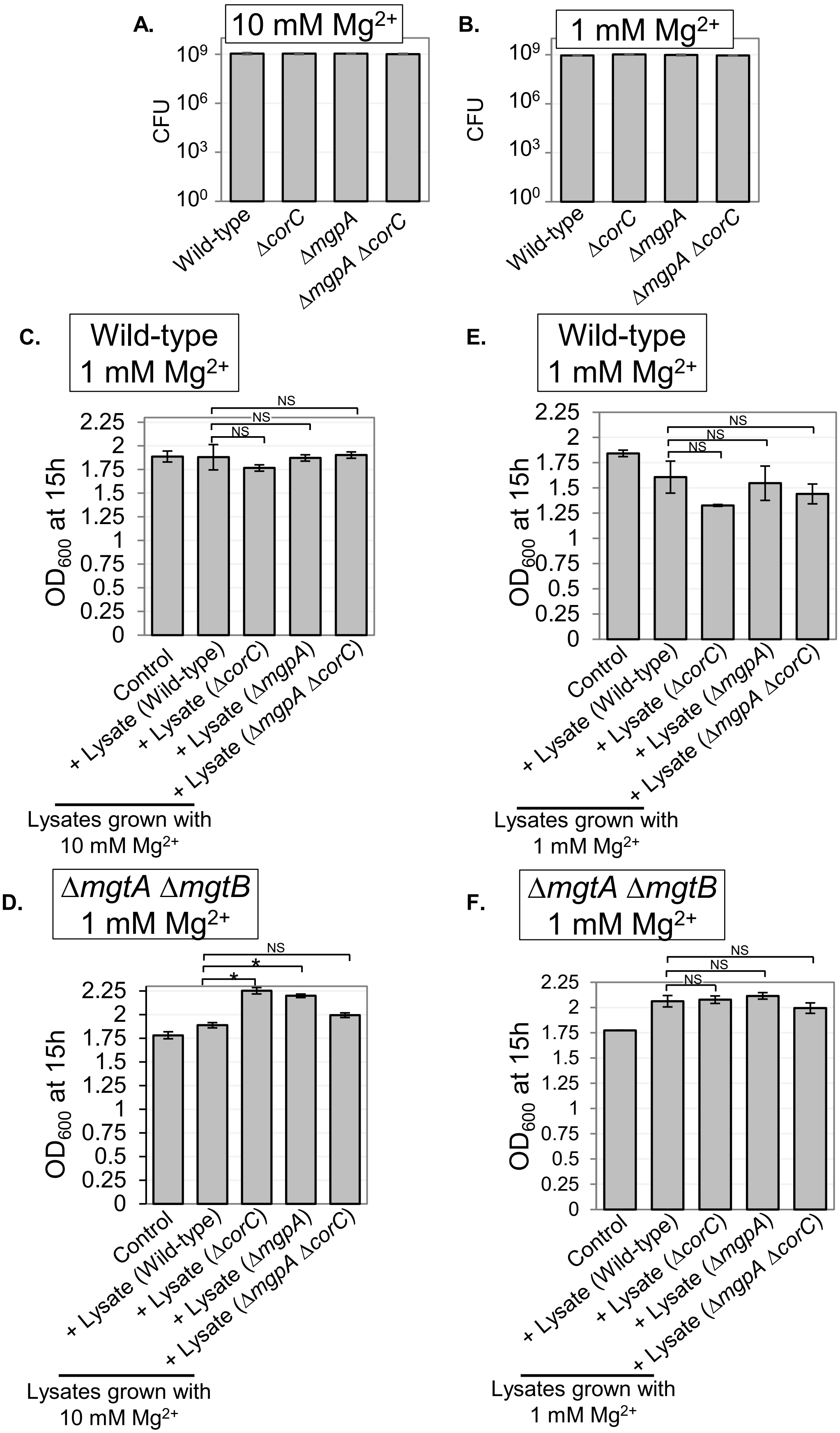

Fig. S9. The lysate from the Δ*mgpA* Δ*corC* strain stimulates growth only comparably to that of the wild-type lysate when the medium contains 1 mM $MgCl_2$. Stationary-phase cultures used for lysate preparation, grown in N-minimal medium (pH 7.4) with (A) 10 mM or (B) 1 mM $MgCl_2$ at 37 ℃, retain full viability. The indicated strains were pre-grown to mid-exponential phase in N-minimal medium (pH 7.4) with 10 mM $MgCl_2$ at 37 ℃, washed, and then diluted into either (C–F) N-minimal medium (pH 7.4) with 1 mM Mg^{2+}, supplemented with 34 μg/mL of each lysate, followed by incubation at 37 ℃. Lysates were prepared from wild-type, Δ*corC*, Δ*mgpA*, and Δ*mgpA* Δ*corC* strains grown in the presence of (C and D) 10 mM or (E and F) 1 mM $MgCl_2$, as described in the Materials and Methods. Final OD_{600} values were measured after 15 hours of incubation. Values are mean ± SD, n = 3. Unpaired t test ($p < 0.05$*, 0.005**, 0.0005***) vs *mgpA*$^+$ *corC*$^+$ parent at the medium. Strains used: 14028, JS2728, JS2729, JS2730, and JS2562.

Table S1. Bacterial strains

Strain[a]	Genotype	Endpoint[b]	Ref[c]
14028	Wild type		
JS2430	Δ*paeA*::Cm		(1)
JS2560	Δ*speED* Δ*speG* Δ*speF* Δ*cadA* Δ*speC* Δ*ldcC* Δ*speB* Δ*speA*::Tc (Δsynth)		(2)
JS2562	Δ*mgtA*::Kan Δ*mgtB*		(2)
JS2563	Δ*speED* Δ*speG* Δ*speF* Δ*cadA* Δ*speC* ΔldcC Δ*speB* Δ*speA*::Tc Δ*mgtA*::Kan Δ*mgtB*		(2)
JS2692	Δ*corC*::Km	732251-733083	
JS2693	Δ*mgpA*::Cm	1937907-1939465	
JS2694	Δ*mgpA*::Cm Δ*corC*::Km		
JS2695	Δ*paeA*		
JS2696	Δ*paeA* Δ*corC*::Km		
JS2697	Δ*paeA* Δ*mgpA*::Cm		
JS2698	Δ*paeA* Δ*mgpA*::Cm Δ*corC*::Km		
JS2699	Wild type + pWKS30		
JS2700	Δ*corC*::Km + pWKS30		
JS2701	Δ*corC*::Km + pWKS30-*corC*		
JS2702	Δ*mgpA*::Cm + pWKS30		
JS2703	Δ*mgpA*::Cm Δ*corC*::Km + pWKS30		
JS2704	Δ*mgpA*::Cm Δ*corC*::Km + pWKS30-*corC*		
JS2705	Δ*mgpA*::Cm Δ*corC*::Km + pWKS30-*mgpA*		
JS2706	Δ*mgpA*::Cm Δ*corC*::Km + pWKS30-*mgpA*-sty291		
JS2707	Δ*yegH*::Cm	2254878-2256450	
JS2708	Δ*yegH*::Cm Δ*corC*::Km		
JS2709	Δ*cvrA*::Cm	1910508-1912225	
JS2710	Δ*cvrA*::Cm Δ*corC*::Km		
JS2711	Δ*corB*::Cm		
JS2712	Δ*corB*::Cm Δ*corC*::Km		
JS2713	Δ*mgtA* Δ*mgtB*		
JS2714	Δ*mgtA* Δ*mgtB* Δ*corC*::Km		
JS2715	Δ*mgtA* Δ*mgtB* Δ*mgpA*::Cm		
JS2716	Δ*mgtA* Δ*mgtB* Δ*mgpA*::Cm Δ*corC*::Km		

JS2717	Δ*speED* Δ*speG* Δ*speF* Δ*cadA* Δ*speC* Δ*ldcC* Δ*speB* Δ*speA*::Tc *corC*::Km		
JS2718	Δ*speED* Δ*speG* Δ*speF* Δ*cadA* Δ*speC* Δ*ldcC* Δ*speB* Δ*speA*::Tc *mgpA*::Cm		
JS2720	Δ*corA*	4171866-4172816	
JS2721	Δ*corA* Δ*corC*::Km		
JS2722	Δ*corA* Δ*mgpA*::Cm		
JS2723	Δ*corA* Δ*mgpA*::Cm Δ*corC*::Km		
JS2724	Δ*paeA* Δ*corC*		
JS2725	Δ*paeA* Δ*mgpA*		
JS2726	Δ*paeA* Δ*corC* Δ*mgpA*		
JS2728	Δ*corC*		
JS2729	Δ*mgpA*		
JS2730	Δ*mgpA* Δ*corC*		
JS2731	Δ*mgpA* Δ*corC* + pWKS30		
JS2732	Δ*mgpA* Δ*corC* + pWKS30-*corC*		
JS2733	Δ*mgpA* Δ*corC* + pWKS30-*mgpA*		
JS2734	att::*corC*60-lacZ	733046-734983	
JS2735	att::*mgpA*24-lacZ	1939442-1940465	

a All *Salmonella* strains are isogenic derivatives of *S. enterica* serovar Typhimurium strain 14028.

b Numbers indicate the base pairs that are deleted (inclusive) as defined in the *S. enterica* serovar Typhimurium 14028 genome sequence (National Center for Biotechnology Information; NC_016856.1)

c This study, unless otherwise indicated

Table 2. Plasmids

Name	Characteristic	Cloned endpoint[a]	Ref[b]
pKD46	*bla* PBAD *gam bet exo* pSC101 *oriTS*		(3)
pCP20	*bla cat* cI857 λPR *flp* pSC101 *oriTS*		(3)
pKD3	*bla* FRT *cat* FRT PS1 PS2 *oriR6K*		(3)
pKD4	*bla* FRT *aph* FRT PS1 PS2 *oriR6K*		(3)
pWKS30	*bla* pSC101 *ori*		(4)
pWKS30-*corC*		732218-733301	
pWKS30-*mgpA*		1937879-1939936	
pWKS30-*mgpA*-sty291		1937879-1939936	
pDX1	*lacZ* tL3 λ*attP oriR6K aacIV tmgB*		(5)
pDX1-*corC*-lacZ		733046-734983	
pDX1-*mgpA*-lacZ		1939442-1940465	

a Numbers indicate the base pairs that are cloned (inclusive) as defined in the *S. enterica* serovar Typhimurium 14028 genome sequence (National Center for Biotechnology Information; NC_016856.1)

b This study, unless otherwise indicated

Table S3. Primers used

Name	Sequence (5' to 3’)	Usage
YIV_pWKS30-12	ATTGCGTTGCGCTCACTGCC	pWKS30-*corC*, pWKS30-*mgpA*, and pWKS30-*mgpA*-sty291
YIV_pWKS30-13	AACGTCGTGACTGGGAAAACC	pWKS30-*corC*, pWKS30-*mgpA*, and pWKS30-*mgpA*-sty291
YIS_73-18	GTTTTCCCAGTCACGACGTTCTATTGCTGTTATTCGTCCAGTTTTG	pWKS30-*corC*
YIS_73-29	GGCAGTGAGCGCAACGCAATATGAGGATCCGTACATTGCC	pWKS30-*corC*
YIS_193-7(RE)	GGCAGTGAGCGCAACGCAATTGCTACCTCCTTTATTATTGTCAACAC	pWKS30-*mgpA* and pWKS30-*mgpA*-sty291
YIS_193-5	GTTTTCCCAGTCACGACGTTCTATAGACTTGATTCCTGCGTG	pWKS30-*mgpA*
YIS_193-6	GTTTTCCCAGTCACGACGTTCCATTAGCGAATTTCCACAGTG	pWKS30-*mgpA*-sty291
YIS_pDX1-1	ATGACCATGATTACGGATTC	pDX1-*corC*-lacZ and pDX1-*mgpA*-lacZ
YIS_pDX1-3	TTGGATCCTCTAGAGTCGACCTGCAG	pDX1-*corC*-lacZ and pDX1-*mgpA*-lacZ
YIS_73-11	CTGCAGGTCGACTCTAGAGGATCCAACCCTACTGAAAAGGCAATAATG	pDX1-*corC*-lacZ
YIS_73-44	GAATCCGTAATCATGGTCATGGAAAAAAATCCCTTTTTAC	pDX1-*corC*-lacZ
YIS_194-9	CTCTAGAGGATCCAACTGACCGTGAATGAGGCGGTC	pDX1-*mgpA*N24-lacZ
YIS_193-26	CGTAATCATGGTCATTGAGGGATCCATTAATAATTCCATGAC	pDX1-*mgpA*N24-lacZ

References

1. Iwadate Y, Ramezanifard R, Golubeva YA, Fenlon LA, Slauch JM. 2021. PaeA (YtfL) protects from cadaverine and putrescine stress in *Salmonella* Typhimurium and *E. coli*. Mol Microbiol 115:1379-1394. https://doi.org/10.1111/mmi.14686
2. Iwadate Y, Golubeva YA, Slauch JM. 2023. Cation homeostasis: coordinate regulation of polyamine and magnesium levels in *Salmonella*. mBio 14:e0269822. https://doi.org/10.1128/mbio.02698-22
3. Datsenko KA, Wanner BL. 2000. One-step inactivation of chromosomal genes in *Escherichia coli* K-12 using PCR products. Proceedings of the National Academy of Sciences of the United States of America 97:6640-6645. https://doi.org/10.1073/pnas.120163297
4. Wang RF, Kushner SR. 1991. Construction of versatile low-copy-number vectors for cloning, sequencing and gene expression in *Escherichia coli*. Gene 100:195-9.
5. Lin D, Rao CV, Slauch JM. 2008. The *Salmonella* SPI1 type three secretion system responds to periplasmic disulfide bond status via the flagellar apparatus and the RcsCDB system. J Bacteriol 190:87-97. https://doi.org/10.1128/JB.01323-07
